# Computational Optogenetics: Empirically-Derived Voltage- and Light-Sensitive Channelrhodopsin-2 Model

**DOI:** 10.1371/journal.pcbi.1003220

**Published:** 2013-09-12

**Authors:** John C. Williams, Jianjin Xu, Zhongju Lu, Aleksandra Klimas, Xuxin Chen, Christina M. Ambrosi, Ira S. Cohen, Emilia Entcheva

**Affiliations:** 1 Department of Biomedical Engineering, Stony Brook University, Stony Brook, New York, United States of America; 2 Department of Physiology & Biophysics, Stony Brook University, Stony Brook, New York, United States of America; 3 Institute for Molecular Cardiology, Stony Brook University, Stony Brook, New York, United States of America; University of Notre Dame, United States of America

## Abstract

Channelrhodospin-2 (ChR2), a light-sensitive ion channel, and its variants have emerged as new excitatory optogenetic tools not only in neuroscience, but also in other areas, including cardiac electrophysiology. An accurate quantitative model of ChR2 is necessary for *in silico* prediction of the response to optical stimulation in realistic tissue/organ settings. Such a model can guide the rational design of new ion channel functionality tailored to different cell types/tissues. Focusing on one of the most widely used ChR2 mutants (H134R) with enhanced current, we collected a comprehensive experimental data set of the response of this ion channel to different irradiances and voltages, and used these data to develop a model of ChR2 with empirically-derived voltage- and irradiance- dependence, where parameters were fine-tuned via simulated annealing optimization. This ChR2 model offers: 1) accurate inward rectification in the current-voltage response across irradiances; 2) empirically-derived voltage- and light-dependent kinetics (activation, deactivation and recovery from inactivation); and 3) accurate amplitude and morphology of the response across voltage and irradiance settings. Temperature-scaling factors (Q_10_) were derived and model kinetics was adjusted to physiological temperatures. Using optical action potential clamp, we experimentally validated model-predicted ChR2 behavior in guinea pig ventricular myocytes. The model was then incorporated in a variety of cardiac myocytes, including human ventricular, atrial and Purkinje cell models. We demonstrate the ability of ChR2 to trigger action potentials in human cardiomyocytes at relatively low light levels, as well as the differential response of these cells to light, with the Purkinje cells being most easily excitable and ventricular cells requiring the highest irradiance at all pulse durations. This new experimentally-validated ChR2 model will facilitate virtual experimentation in neural and cardiac optogenetics at the cell and organ level and provide guidance for the development of *in vivo* tools.

## Author Summary

Optogenetics, the use of light-sensitive ion channels for stimulation of mammalian cells and tissues, offers specificity and superior precision of control compared to traditional chemical or electrical means of stimulation. In particular, Channelrhodospin-2 (ChR2), a light-sensitive ion channel, originally derived from algae, has found wide-spread application in neuroscience for controlled stimulation of different brain regions. More recently, this work was extended to other organs, including the heart, where it opens the possibility for a new generation of optical pacemakers. The development of new optogenetic tools that allow for more efficient optical stimulation can be guided by computational prediction of the response of different cells and tissues to light. In this report, we provide a new computational model of ChR2 that was empirically validated and can be inserted into different cell types – neurons or heart cells – for virtual optical stimulation and prediction of optimal light-delivery arrangements, minimum energy needs etc. Overall, virtual optogenetics can accelerate the development of new optical stimulation tools for better understanding and control of brain and heart function.

## Introduction

Quantitative biophysical approaches have strong traditions in neural and cardiac electrophysiology [1]–[4]. The dynamic and highly nonlinear process of cellular excitation is still successfully captured today by Hodgkin and Huxley's empirical modeling framework from 1952 [4], even though the complexity of computational models is ever-increasing to reflect new discoveries about diversity in ion channels and their characteristics. The function of classical ion channels is described by voltage- and time-dependent equations; in some cases, additional chemical or mechanical control parameters are used. Only recently, *light-sensitive ion channels* originally found in bacteria or yeast have proven relevant to mammalian electrophysiology [5], [6]. These ion channels provide alternative (optical) means of excitation with superior (cell type) specificity and spatiotemporal resolution compared to electrical stimulation. A new field of genetically expressing light-sensitive ion channels in vertebrates to confer specific optical responsiveness has become known as optogenetics [7]. Computational optogenetics, i.e. quantitative modeling for virtual experimentation in optogenetics, is in its infancy with only a handful of published reports [8]–[12]. Computational optogenetics can aid in quick (virtual) testing of the behavior of newly developed tools in a wide range of cell types, and within realistic tissue settings. Such simulations can also drive the rational design of new optogenetics tools optimized for a specific cell/tissue environment. Furthermore, they can assist in the proper interpretation of experiments within a complex cell/tissue environment, which is often ambiguous and challenging.

Channelrhodopsin-2 (ChR2) is the first light-sensitive ion channel that, after its cloning in 2003 [13], found widespread use as an optical actuator in neuroscience [5], [14]–[16], and more recently - in cardiac applications [10], [17]–[19]. ChR2 is both light- and voltage-sensitive. The conducting pore of the channel associates (via a covalent bond) to retinal, which serves as the chromophore (the light-sensing element). Interaction of all-trans-retinal with a photon of the proper wavelength (470 nm) leads to instantaneous isomerization to 13-cis-retinal. This transition triggers the opening of the ion channel allowing cation movements down their electrochemical gradient, with preferential selectivity to H^+^ [13], [20]. ChR2 provides exclusively inward current (at negative membrane potentials) with a reversal potential near 0 mV. The single channel conductance for the wild type ChR2 is small compared to classical excitatory ion channels (e.g. sodium channels) with reported values ranging from 40–90 fS [13], [21], [22] to 0.25–2.42 pS [20]. Genetically engineered mutants of ChR2, e.g. H134R [6], T159C [23] and ET/TC [23], offer augmented excitatory current; while other mutants alter kinetics to serve different uses, e.g. fast ChETA [24] or stable switches with a prolonged open state [25].

As a prototypical opsin, ChR2 has become the focus of the initial efforts to create a computational branch of optogenetics. ChR2's photocycle has been studied extensively and it formed the basis for abstracting the behavior of the channel to Markov type (three- and four-) state models proposed recently by Hegemann [12] and Nikolic [9]. Versions of a four-state model are currently favored based on experimental evidence for four kinetic intermediates with differing time constants [12], [26], [27]. Current efforts also include modeling of different mutants [8], [11], as well as the integration of ChR2 into comprehensive cell models – neurons [8], [11], [28] and cardiomyocytes [10], [29]. While ChR2's light sensitivity has been studied extensively [13], data on its voltage dependence are limited [30]. Early models [9], [11] assumed a linear current-voltage relationship and no voltage-dependent gating. Since experimental results demonstrate prominent inward rectification [6], [18], [20], [21], [30], [31], more recent ChR2 models have incorporated some nonlinearity [8], [10]. Voltage sensitivity, however, has yet to be comprehensively addressed.

Proper quantitative prediction of the functionality of light-sensitive ion channels, including ChR2, within mammalian cells and tissues, requires that the kinetics and amplitude of the observed current are scaled to reflect physiological conditions, even when the channel is characterized primarily at room temperature. In this study, we provide a rigorously validated quantitative model of a mutant of ChR2 with enhanced current, the H134R variant, based on experimental data collected over a wide range of irradiances and voltages. The result is a new ChR2 model with updated voltage dependences – not only of the current-voltage response but also of relevant kinetic parameters. We also empirically derive scaling factors (Q_10_) to adjust processes to physiological temperature. Experimental validation of the model-predicted ChR2 behavior in guinea pig ventricular cardiomyocytes was done using an optical action potential clamp. Finally, our simulations with different human cardiomyocyte types (ventricular, atrial and Purkinje cells) offer insights into the differential cell-specific response to ChR2-mediated stimulation depending on the ion channel milieu and about actual light irradiances needed to excite such human cells. This validated ChR2 model can serve as a new tool for expanding computational optogenetics to complex tissues and organs, e.g. the brain and the heart.

## Results

We developed and validated a new mathematical model of the light-sensitive ion channel ChR2 (with the H134R mutation) based on a comprehensive experimental data set collected in a ChR2(H134R)-HEK293 stable cell line we have described earlier [18]. This new model uses a previously established state-transition diagram for the functioning of the channel but yields some new voltage and light dependencies that may be critical in understanding the operation of ChR2 in cardiomyocytes and other cells with more complex action potential morphology.

### Using empirical constraints for model development

**Figure 1:**
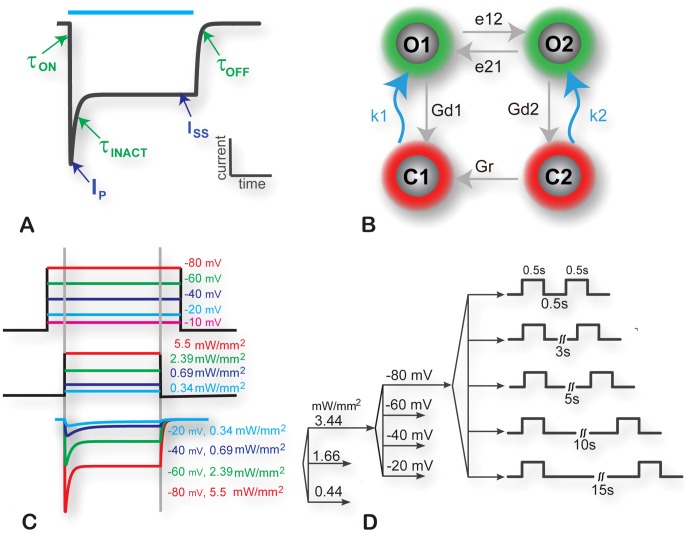
Experimental protocols, empirical measures and construction of ChR2 model. **A**. Empirical measures extracted from experimental measurements and used to constrain/optimize the model. These are five parameters capturing the amplitude and kinetics of ChR2 current: peak current, I_P_; steady-state current, I_SS_, activation time constant, τ_ON_; inactivation time constant, τ_INACT_; and deactivation time constant, τ_OFF_; they were quantified for each cell under all described experimental conditions. Blue bar indicates light pulse duration. **B**. ChR2 model structure used in this study, adapted from [9], [12], with two closed states (C_1_ and C_2_) and two open states (O_1_ and O_2_), seven transition rates, of which two (k_1_ and k_2_) are light-dependent; see Table 1 for details. **C**. Experimental protocol with voltage clamp and optical pulse application for a total of 20 combinations per cell: 5 holding voltages ranging from −80 to −10 mV and 4 irradiances ranging from 0.34 to 5.5 mW/mm^2^. Example traces (ChR2 current) are shown for selected 4 (out of 20) combinations. **D**. Recovery-from-inactivation experimental protocol (S1–S2 pulse protocol) for a total of 60 combinations of conditions per cell (3 irradiances, 4 holding voltages and 5 inter-pulse intervals), as indicated.

To construct a ChR2 model that matches ion current responses across voltages and irradiances, we defined five empirical measures of the ChR2 current response to a light pulse (**Figure 1A**): two amplitude measures (peak current, I_p_, and steady-state current, I_ss_) and three kinetic measures (time constant of activation, τ_ON_, of deactivation, τ_OFF_, and of inactivation, τ_INACT_). These five simplified morphology-capturing measures were quantified (via nonlinear fitting described in the Materials and Methods section) for experimental traces obtained under various sets of voltages and irradiances (**Figure 1C**). They were then used as guidance/constraints for the fitting of the actual model parameters, including the seven state transition rates in **Figure 1B**, assuming a four-state model structure, as previously proposed [9], [12]. Fitting was done using multi-parameter optimization via simulated annealing in MATLAB, Mathworks, Natick, MA (see Materials and Methods). Additional constraints were imposed by data on recovery from inactivation obtained using a classic S1–S2 protocol (**Figure 1D**), yielding a sixth empirical measure – time constant of recovery from inactivation, τ_R_.

**Figure 2:**
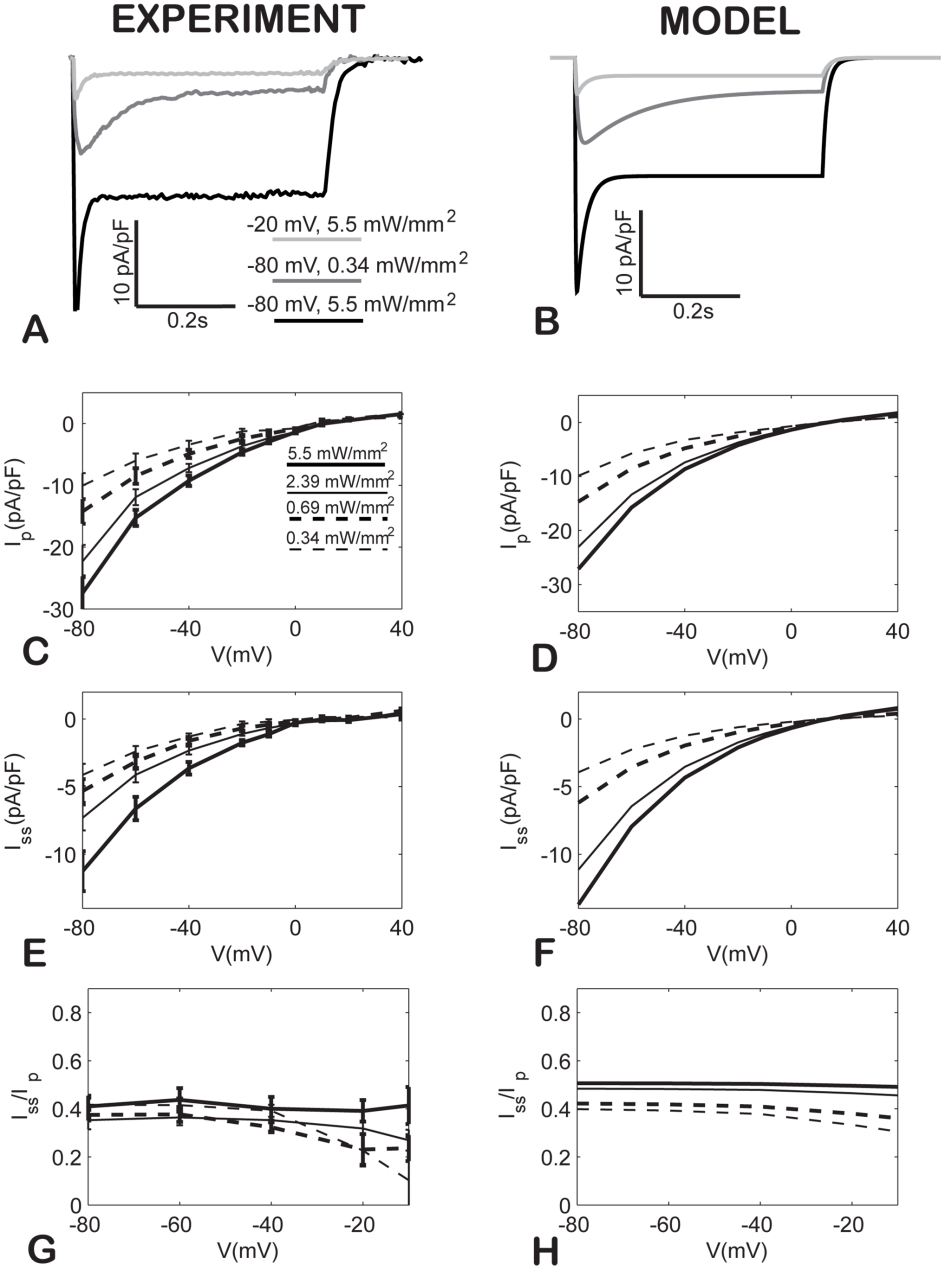
Inward current-voltage rectification for ChR2 in experiments (left) and in the model (right). Experimental (**A**) and model (**B**) example traces for ChR2 current in response to 0.5 s light pulses, 470 nm, at specified irradiances and holding voltages. **CD**. Current-voltage (I–V) curves for the peak current (I_P_). **EF**. Current-voltage (I–V) curves for the steady-state current (I_SS_). **GH**. Ratio of I_SS_/I_P_ as a function of voltage. Experimental data in **C, E**, and **G** are presented as mean±S.E., n = 7.

We assumed mono-exponential processes for the respective transitions, although it has been suggested that this may not be completely accurate for the deactivation (τ_OFF_) of wild type ChR2 [9]. Nevertheless, for our experimental data on the H134R mutant, the mono-exponential curve fits yielded a good approximation and faithfully captured the magnitude and morphology of the ChR2 current under various experimental conditions (**Figure 2AB**). When bi-exponential fits were enforced for some of the transient processes, as suggested in [9], these degenerated to mono-exponential curves for our experimental traces.

### Accurate capture of ChR2's prominent inward current-voltage rectification

We and others have shown that ChR2 and its variants display strong inward rectification for both the peak and steady-state current at voltages above 0mV [18], [20], [30]. This non-linear voltage dependence has been omitted in most prior models [9], [11], and in general, ChR2's voltage dependence has not been accurately represented in previous quantitative descriptions. Therefore, we collected a comprehensive experimental data set using multiple combinations of voltage and irradiances. We then performed multiparameter optimization on the experimental data to derive an empirical rectification function (see **Table 1** and **Figure S1**), and reproduce the experimental I–V curves with the model (**Figure 2C–H**). Experimental measurements of the reversal potential of ChR2 with high precision are challenging because of the small current and poor signal-to-noise ratio around that point. For the model, we assumed a reversal potential of zero, which is consistent with our measurements (variations within ±5 mV), data by others [13], [30], and is within theoretical estimates considering typical intra- and extracellular concentrations and data on ChR2 selectivity to H^+^, Na^+^, K^+^ and Ca^2+^ from [20].

**Table 1:** Model parameters.

param	units	definition	value	used by others	ref	notes
V	mV	membrane voltage	var	var		
E_ChR2_	mV	reversal potential for ChR2	0	−5– +10		measured value was close to 0 mV
g_ChR2_	mS/cm^2^	max conductance (scaling)	0.4	0.75	[11]	scaling conductance
G(V)	-	voltage-dependent rectification function	[10.6408–14.6408* exp(-V/42.7671))]/V	(1-exp(-V/40))/(V/15)	[8], [10]	derived from experimental I–V curves to capture rectification
				0.05V^2^−0.0692V+9.442		
γ	-	ratio of conductances of O_2_/O_1_	0.1	0.05–0.1	[8], [9], [11]	
O_1_, O_2_	-	open state probabilities	var (0 to 1)	var (0 to 1)	[12]	
C_1_, C_2_	-	closed state probabilities	var (0 to 1)	var (0 to 1)	[12]	
G_d1_	ms^−1^	rate constant for O_1_→C_1_ transition	0.075+0.043 *tanh((V+20)/−20)	0.084;.0.108; 0.11	[11], [12]	voltage dependence was introduced to fit the experimental data
G_d2_	ms^−1^	rate constant for O_2_→C_2_ transition	0.05	0.1254; 0.036; 0.025	[11], [12]	
G_r_	ms^−1^	rate constant for C_2_→C_1_ transition	4.34587 * 10^5^ * exp(−0.0211539274*V)	0.004; 0.0004	[11], [12]	voltage dependence was introduced to fit experimental S1–S2 recovery data
e_12_	ms^−1^	rate constant for O_1_→O_2_ transition	e_12d_ = 0.011	e_12d_ = 0.011; 0.0297; 0.03; 0.0	[8], [9], [11], [12]	
			e_12_ = e_12d_+c_1_*ln(1+I/c_2_)	e_12_ = e_12d_+c_1_*ln(1+I/c_2_)		
			I – irradiance	I – irradiance		
			c_1_ = 0.005; c_2_ = 0.024	c_1_ = 0.005; c_2_ = 0.024		
e_21_	ms^−1^	rate constant for O_2_→O_1_ transition	e_21d_ = 0.008	e_21d_ = 0.008; 0.018; 0; 0.015	[8], [9], [11], [12]	
			e_21_ = e_21d_+c_1_ *ln(1+I/c_2_)	e_21_ = e_21d_+c_1_*ln(1+I/c_2_)		
			I – irradiance;	I – irradiance;		
			c_1_ = 0.004; c_2_ = 0.024	c_1_ = 0.004; c_2_ = 0.024		
k_1_	ms^−1^	light-sensitive rate constant for C_1_→O_1_ transition	φ_1_(F, t) = ε_1_F*p = var	φ_1_(F, t) = ε_1_F*p = var	[11], [12]	
				0.369 (at 5 mW/mm^2^)		
k_2_	ms^−1^	light-sensitive rate constant for C_2_→O_2_ transition	φ_2_(F, t) = ε_2_F*p = var	φ_2_(F, t) = ε_2_F*p = var	[11], [12]	
				0.369 (at 5 mW/mm^2^)		
ε_1_	-	quantum efficiency for photon absorption from C_1_	0.8535	0.8535; 0.5	[11]	
ε_2_	-	quantum efficiency for photon absorption from C_2_	0.14	0.14; 0.025; 0.15	[11]	
σ_ret_	m^2^	absorption cross-section for retinal	12×10^−20^	1.2×10^−20^	[12]	reflects light sensitivity; this parameter was increased to match Iss/Ip data
λ	nm	wavelength of max absorption for retinal	470	470		
I	mW/mm^2^	Irradiance	var (typically 0 to 10)	var (typically 0 to 10)		
w_loss_	-	scaling factor for losses of photons due to scattering or absorption	0.77 (1.3)	0.77 (1.3); 0.9 (1.1)	[9]	can be defined as division (or multiplication) factor
F	ms^−1^	photon flux: number of photons per molecule per second	σ_ret_*I/(E_ph_*w_loss_) = (*σ*_ret_/hc)*Iλ/*w*_loss_ = 0.0006*Iλ/w_loss_	σ_ret_*I_c_/(E_ph_*w_loss_) = (*σ*_ret_/hc) *Iλ/*w*_loss_ = 0.00006*Iλ/w_loss_	[9], [11]	order of magnitude mistakes in unit conversion in [11], [12]
p, S_0_(θ)	-	state-variable, time- and irradiance-dependent activation function for ChR2 (post-isomerization)	dp/dt = (S_0_(θ_optical_)−p)/(τ_ChR_)S_0_(θ) = 0.5 (1+tanh(120(θ−0.1)))*-*	same as in [11]; the inflection point of the sigmoidal S-function is low (0.1 mW/mm^2^)	[9], [11]	time-dependent function reflecting the probabilistic, non-instantaneous response of the ChR2-retinal complex to light
τ_ChR2_	ms	time constant of ChR2 activation	1.3	1.3	[9]	
θ	mW/mm^2^	optical stimulation protocol (irradiance)	100*I	var (typically 0 to 1000)	[11]	
hc	kg m^3^/s^2^	product of Planck's constant and the speed of light	1.986446×10^−25^	1.986446×10^−25^		

The ratio of the steady-state to peak current for ChR2 varies considerably among its genetically-engineered variants [16], [32]. Consequently, a proper representation of this parameter over the matrix of voltage-irradiance values required reconsideration of the light sensitivity of the current, and adjustment of some parameters, especially an increase in the absorption cross-section, σ_ret_. This parameter is directly dependent on the extinction coefficient for retinal and its value is spectrally sensitive [33].

### Differential light- and voltage-dependence of ChR2's kinetic parameters (activation, deactivation and inactivation)

**Figure 3:**
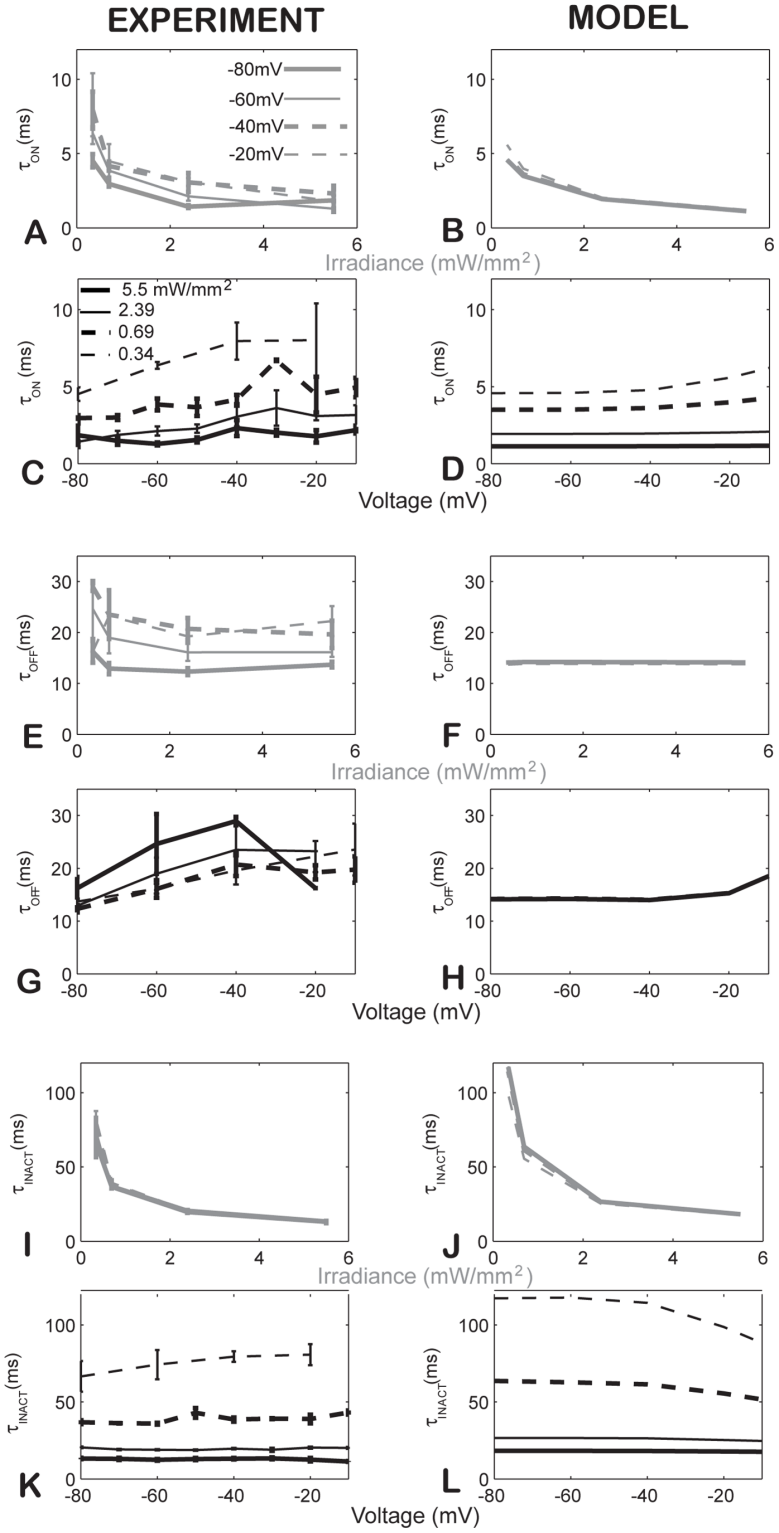
Light- and voltage-dependence of kinetic parameters in experiments (left) and in the model (right). **AB**. Light dependence of τ_ON_ across four voltage values. **CD**. Voltage dependence of τ_ON_ across four irradiance levels. **EF**. Light dependence of τ_OFF_ across four voltage values. **GH**. Voltage dependence of τ_OFF_ across four irradiance levels. **IJ**. Light dependence of τ_INACT_ across four voltage values. **KL**. Voltage dependence of τ_INACT_ across four irradiance levels. Experimental data in **A, C, E, G, I**, and **K** are presented as mean±S.E., n = 5.

None of the previously published ChR2 models include voltage–dependent kinetics. Our experimental data revealed strong non-linear light dependence for τ_ON_ and τ_INACT_ (exponentially decreasing with higher irradiance), which was statistically significant (P<0.001) in both cases, but a relatively light-insensitive τ_OFF_, P = 0.044 (**Figure 3A–I**). Furthermore, we show experimentally (consistent with prior data [30]) that τ_INACT_ is completely voltage-independent (P = 0.178), but τ_ON_ and τ_OFF_ exhibit mild voltage dependence, which was statistically significant (P = 0.009 and P<0.01, respectively), not included in any previous models. The latter was captured in the model by introducing voltage dependence in the transition from O_1_ to C_1_ (G_d_), see **Table 1** **, Figure S1**. The model offers a quantitative match for these key kinetic parameters across the voltage-irradiance matrix, **Figure 3**.

### Recovery from inactivation is a voltage- and light-dependent process

**Figure 4:**
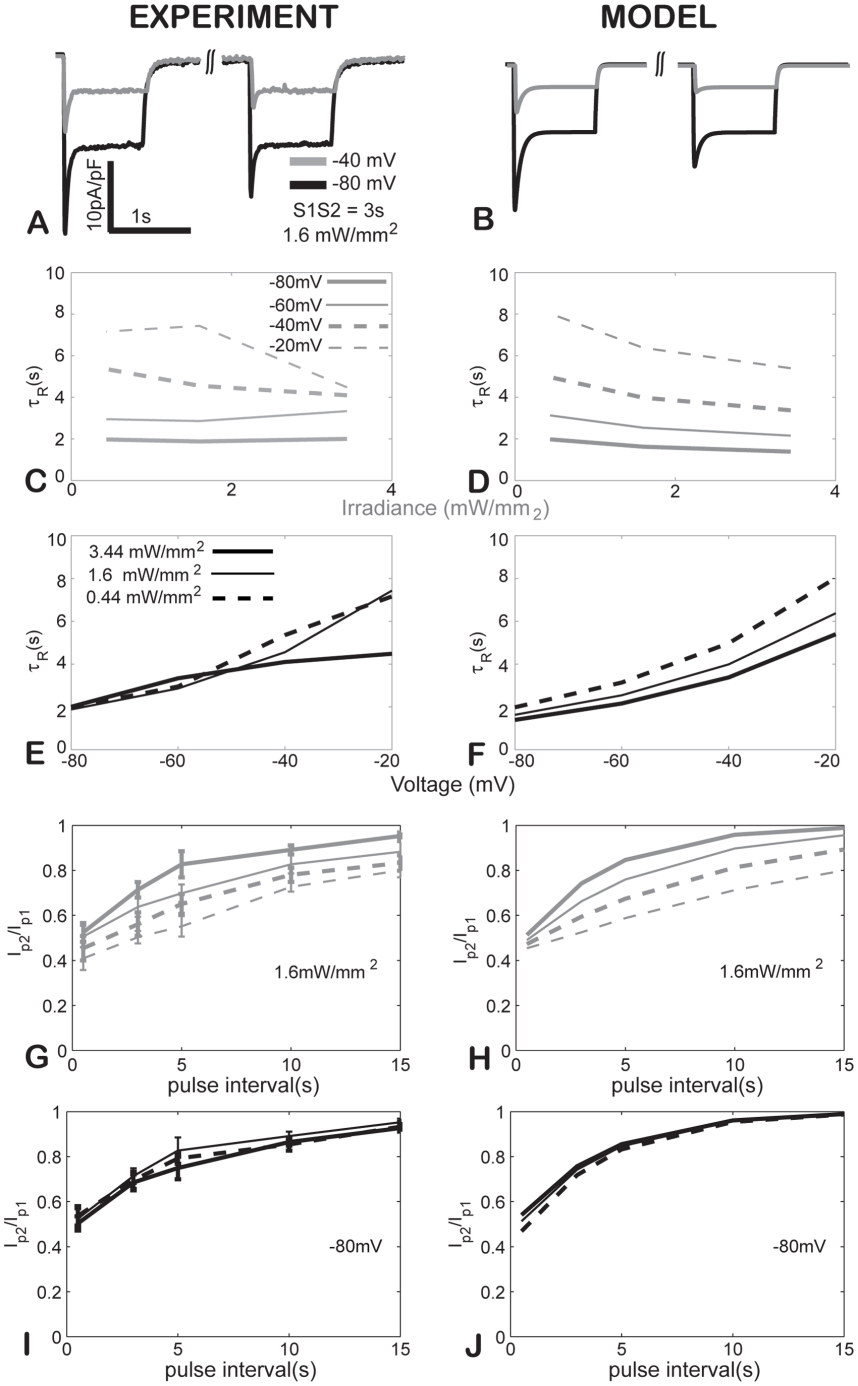
Kinetics of recovery from inactivation for ChR2 in experiments (left) and in the model (right). **AB**. Experimental and model traces in response to S1–S2 protocol, 3 s inter-pulse interval, irradiance of 1.6 mW/mm^2^ and holding voltages of −40 and −80 mV. **CD**. Light dependence of τ_R_ across four voltage values. **EF**. Voltage dependence of τ_R_ across three irradiance levels. **GH**. Ratio of the peak currents in response to S2 and S1, I_P2_/I_P1_, as function of inter-pulse interval, across four voltage values. **IJ**. Ratio of the peak currents in response to S2 and S1, I_P2_/I_P1_, as function of inter-pulse interval, across three irradiance levels. Experimental data in **C** and **E** were fits to the average curves, n = 4. Experimental data in **G** and **I** are presented as mean±S.E., n = 4.

Recovery from light-induced inactivation was studied under an S1–S2 protocol (**Figure 1D**). Sample responses (an experiment and model simulation) illustrate that for an inter-pulse interval of 3 s, a fixed irradiance (1.6 mW/mm^2^) and two holding voltages (−40 mV and −80 mV), the recovery is incomplete, **Figure 4A–B**. A rigorous experimental examination of the recovery from inactivation revealed that the process (τ_R_) depends on both voltage and irradiance (**Figure 4C–J**), where more positive voltages slow the recovery significantly, while higher irradiances mildly speed the recovery from inactivation. Since in the model, the C_2_ to C_1_ transition (G_r_) is the parameter most directly linked to τ_R_, we derived an empirical voltage dependence of this rate constant matching the experimental findings. A qualitative summary of the voltage- and irradiance dependence of the six key parameters describing the ChR2 response is given in **Table 2**.

**Table 2:** Light- and voltage-dependence of key ChR2 characteristics.

ChR2 characteristic	Irradiance dependence	Voltage dependence
*τ*_ON_	↓ (strong nonlinear)	↑ (weak)
*τ*_INACT_	↓ (very strong nonlinear)	∼
*τ*_OFF_	∼	↑ (weak)
I_p_	↑ (nonlinear)	↓ (highly nonlinear, inward rectification)
I_ss_	↑ (slightly nonlinear)	↓ (highly nonlinear, inward rectification)
I_ss_/I_p_	↑ (nonlinear)	↓ (weak)
*τ*_R_	↓ (weak)	↑
I_p2_/I_p1_	∼	↓
I_s2_/I_s1_	∼	∼
E_ChR2_	↑ (exp data available)	NA

Note: arrows (↑↓) indicate direction of change of a parameter with increasing irradiance or increasing (more positive) voltage, ∼indicates lack of sensitivity.

### ChR2 current is temperature-dependent: Q_10_ derivation for scaling to physiological temperatures

**Figure 5:**
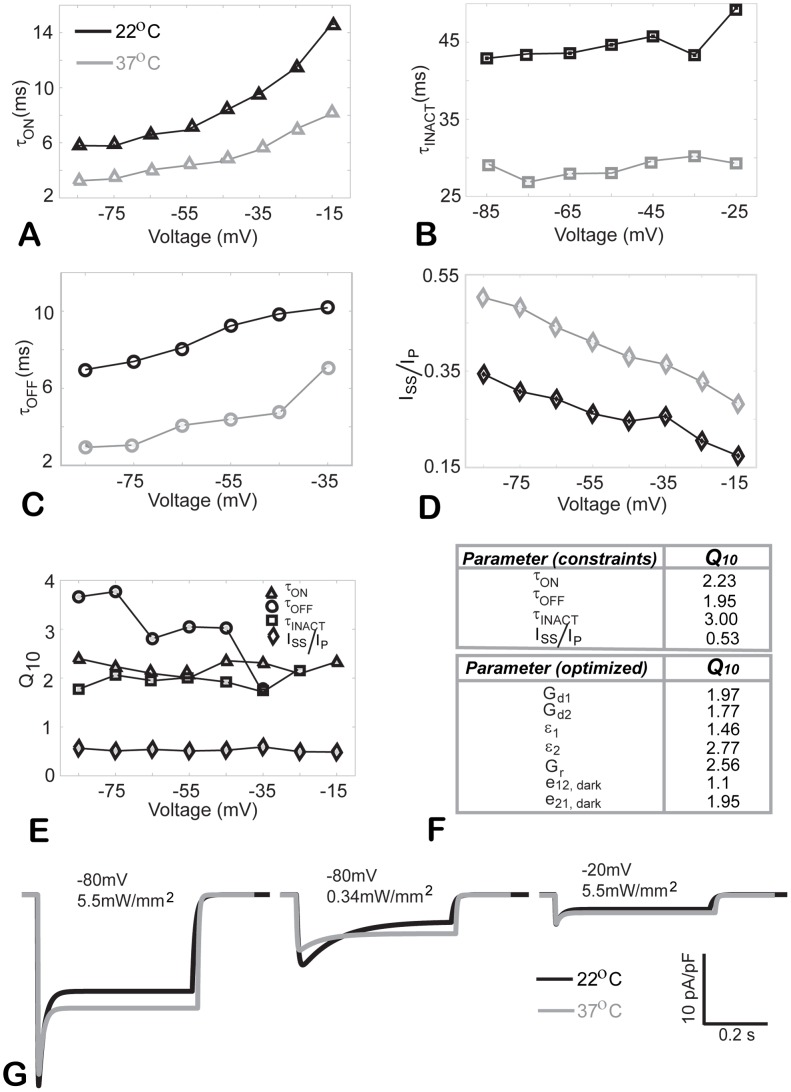
Temperature dependence of ChR2 current. **A–D** Experimental data, adapted from [30], showing the effect of temperature (22°C and 37°C) on τ_ON_, τ_INACT_, τ_OFF_ and I_SS_/I_P_ for a range of voltages. **E**. Temperature scaling factors (Q_10_ values), derived from the data in **A–D**. **F**. Summary of average Q_10_ values (from **E**) used as constraints for the model optimization; optimized Q_10_ values for the rate parameters of the model. **G**. Example model traces of ChR2 current for the specified voltage and irradiance values at 22°C and 37°C.

As with the majority of studies on ion channels, our experiments were conducted at room temperature. In order to obtain a ChR2 model suitable for simulations under physiological conditions (37°C) and insertion into cardiac (or other) cell and tissue models, we sought to derive and implement temperature scaling factors, commonly known as Q_10_'s. Experimental data from [30], at 22°C and 37°C, shown in **Figure 5A–D**, were used to derive Q_10_ values for our three main kinetic parameters and the Iss/Ip ratio. These Q_10_ values were found to be relatively voltage-independent, except for τ_OFF_, and within the range of Q_10_ values for classic ion channel parameters [34], **Figure 5E–F**. The experimentally derived Q_10_ values were used as constraints in the nonlinear optimization procedure introducing temperature scaling of the model rate constants (**Figure 1B**) to yield the values listed in **Figure 5F**. Representative current traces from the model for 22°C and 37°C are shown in **Figure 5G**. In general, elevated temperature results in faster kinetics and a larger fraction of sustained current Iss, as seen experimentally [30].

Optical stimulation can affect local temperature experienced by the target cells. However, for the wavelengths considered here (470 nm) and the irradiance levels typically used in our experiments (0.1 to 5.5 mW/mm^2^), we find by theoretical analysis that such temperature changes will be negligible. More specifically, using a closed-form solution for the diffusive partial differential equation to model maximum temperature change during heating of a water-based particle within aqueous medium similar to [35] (see **Text S1**), we find that after a single 90 ms light pulse at 5.5 mW/mm^2^, temperature at the center of the light beam will rise by approximately 0.013°C, and for much longer pulses at this irradiance, the saturation temperature rise will be less than 0.04°C. Therefore, heating effects due to the light application itself were not taken into account in the model.

### Functionality of the ChR2 within different cardiac cell types – implications for optical excitability

**Figure 6:**
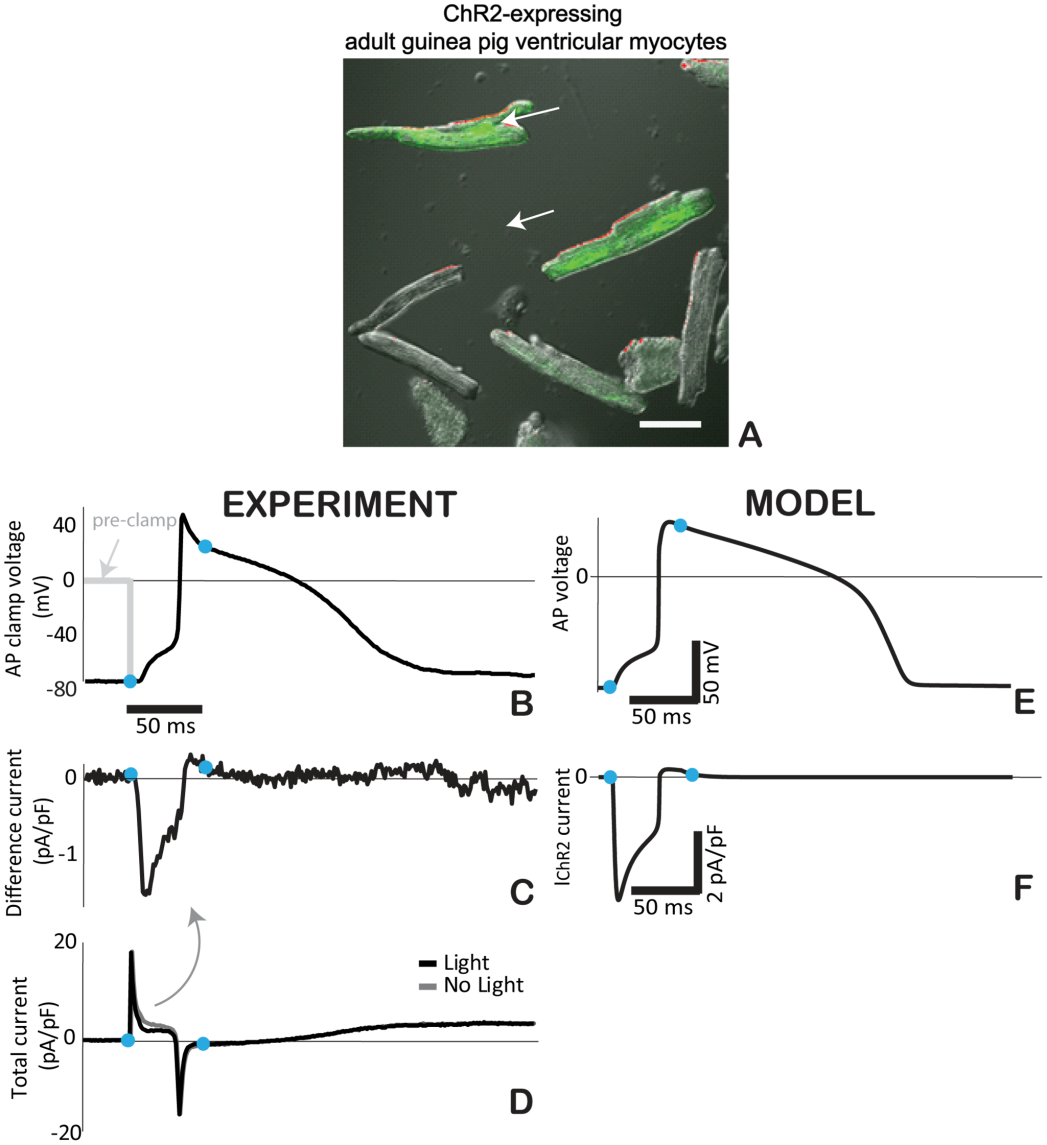
ChR2 current during the cardiac action potential via AP clamp. **A**. Adult guinea pig ventricular cardiomyocytes after 48 h of viral infection with Ad-ChR2(H134R)-EYFP, green fluorescence indicates ChR2 expression; scale bar is 50 µm. Experimental (**B–D**) and modeling (**E–F**) traces for guinea pig ventricular cells. **B**. Optically-triggered action potential (50 ms pulse at 470 nm, 1.5 mW/mm^2^) used for the AP clamp; dotted line indicates the voltage clamp conditions upon application of the waveform; blue dots indicate the beginning and end of the optical pulse. **C**. Extracted I_ChR2_ as the difference current from the total current traces (panel **D**) recorded in dark conditions and with a light pulse. **E**. Analogous optically-triggered action potential in a model of a guinea pig ventricular cell. **F**. The underlying I_ChR2_ according to the model.

There are no prior records of the behavior of I_ChR2_ during a cardiac action potential. To obtain such data, adult guinea pig ventricular myocytes, virally transduced with ChR2, were used here. A modified version of the action potential clamp (AP clamp) [36] was applied (see Materials and Methods). During the voltage clamp with an optically-triggered AP, the absence of light (dark condition) was used as a selective ChR2 “blocker”, while the ChR2 “enabling” condition required a precisely-timed optical pulse during the AP clamp. An example of such experimental record is shown in **Figure 6B–D**, confirmed in additional three cells. When timing of the optical pulse was altered (delayed by 100 ms), the distinctive inward net current was lost, thus corroborating ChR2 as a likely source. Striking morphological similarity, albeit not exact quantitative match, is seen between the experimentally-obtained and model-simulated ChR2 current within the ventricular action potential of a guinea pig cell (**Figure 6**). Since the I_ChR2_ scaling in the model is based on average cell data in a HEK-ChR2 cell line, some difference in the magnitude of the current between the model and an individual guinea pig ventricular myocyte, likely with different ChR2 expression levels, is not surprising.

**Figure 7:**
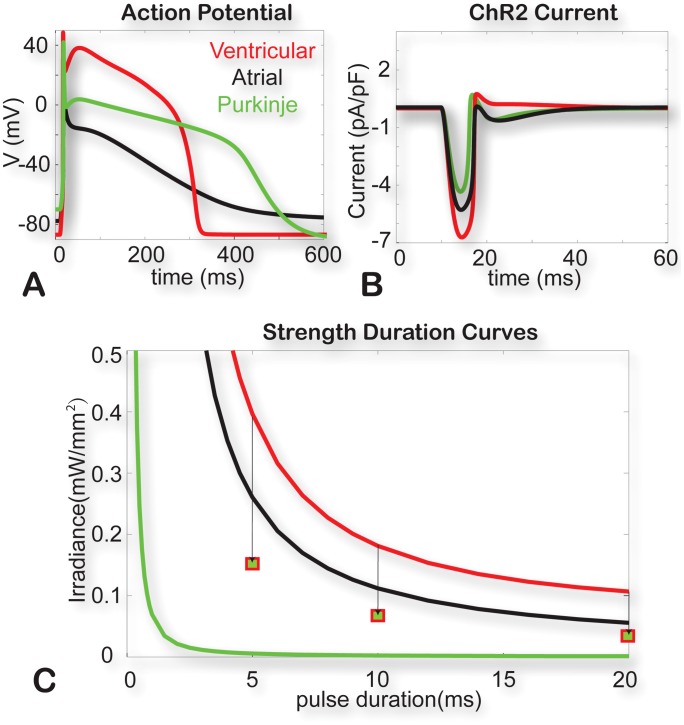
Optical excitation in human cardiac cell types. **A**. Optically-triggered action potentials (10 ms pulse at 470 nm, 0.5 mW/mm^2^) in human ventricular, atrial and Purkinje cells. **B**. Underlying ChR2 current upon the action potential generation for the three cell types. **C**. Strength-duration curves for the three cell types. Squares show simulated values for optical excitation threshold in ventricular myocytes with a Purkinje formulation of I_K1_.

The final ChR2 model, scaled to physiological temperatures, was inserted into several other cardiac cell types, including a human ventricular myocyte [37], human atrial myocyte [38], and a human Purkinje cell [39], **Figure 7**. In the dark, the channel has no contribution to the cell electrophysiology. Upon a typical supra-threshold light pulse (in this example, 10 ms pulse at 470 nm, 0.5 mW/mm^2^), an inward current is generated, that is sufficient to initiate an action potential in each of the cells (**Figure 7A**). This light-triggered current is self-terminating – a slight outward current is generated after the membrane potential exceeds the reversal potential for ChR2. It is interesting to note the differential response of ChR2 within these three cell types - a smaller ChR2 current is sufficient to bring about excitation in Purkinje, while ventricular myocytes require the highest current, i.e. are the hardest to excite by light across a range of pulse amplitudes and durations (strength-duration curves shown in **Figure 7C**). Identical conductance values were chosen for ChR2 in all three cell types based on our data from the ChR2-HEK cell line; g_ChR2_ may vary depending on the efficiency of gene expression and the specific cell environment. We find that ChR2 current density or expression levels will scale the strength-duration curves in a similar way for all three cell types, i.e. higher expression levels will monotonically reduce the optical energy needed to excite (**Figure S2**).

Longer-term fast optical pacing will produce partial inactivation of ChR2 current by shifting the state occupancy towards the lower-conductance light-adapted states (O2 and C2). For example, a pacing sequence delivered to a human ventricular myocyte at 2 Hz 10 ms optical pulses results in about 22% reduction in the peak available ChR2 current after 100 sec of stimulation, **Figure S3**.

## Discussion

### Motivation for developing a new ChR2 (H134R) model

The quantitative modeling of light-sensitive ion channels from bacteria or yeast, e.g. ChR2, recently used for functional control of mammalian cells and tissues, forms the foundation of a new field - computational optogenetics, [8]–[12]. The first computational models were designed to recapitulate primarily the light-responsiveness in optical stimulation of neurons mediated by ChR2 [9], [12], introducing only rudimentary linear voltage dependence. In contrast to its neuronal use, functionality of ChR2 within cardiomyocytes is impacted by the morphology of the longer action potentials, and therefore voltage dependences need to be described accurately. **Figure S4** compares the response of a model without nonlinear voltage dependences to the model presented here. Without nonlinearity in the current-voltage characteristics (i.e. no rectification), the functionality of the ion channel within a cardiac cell is misrepresented by an erroneous repolarizing current during the plateau phase of the action potential (**Figure S4C**). Our motivation was to develop a model that accurately captures features of the channel that can affect its action within cardiomyocytes, especially as related to voltage-dependent properties.

Recently, a three-state ChR2 model was published [10] that focuses on the same mutant as this study – H134R. The model derived here, matching key empirical measures in our comprehensive experimental data, **Figures 1** **–** **4**, was compared to the published three-state model of ChR2-H134R. **Figures S5–S6** reveal that this prior model was inconsistent with our data. The main differences include a smaller steady-state current in the published model (**Figure S5**), much lower steady-state to peak ratios (**Figure S6A–B**), and slower opening of the channel across irradiances and voltages (**Figure S6C–D**). These significant differences in the response to light and voltage could lead to inaccurate predictions of the action of the channel within cardiomyocytes as shown in **Figure S7**. The source of this discrepancy is unclear but potential confounding factors may include the different cell environment - we used a homogeneous experimental data set acquired in stable HEK cell line with ChR2-H134R, while the published model was partially based on data obtained in cardiomyocytes derived from embryonic stem cells expressing this channel. While classical voltage-gated ion channels are often characterized in artificial heterologous systems under the assumption that their salient features remain independent of the cell environment, for the light-sensitive ChR2 that associates with a cell-derived chromophore, all-trans-retinal, this assumption will need to be tested. To this end, there is no systematic evidence for cell-dependent properties of ChR2 or any other optogenetic tools. Another possible contributor to the discrepancy between the two models in **Figures S5–S7**, can be insufficient constraints used in the prior model construction, i.e. the comprehensiveness of the matrix of voltages and irradiances employed previously was unclear. An interesting question, stemming from the discrepancy between these two mathematical models, is whether the assumed state-transition structure (three states vs. four states in our model) may have been restrictive in any way.

### Model structure and empirical approach to ChR2 modeling

Following a tradition of modeling classical ion channels, we adopted an empirical approach towards modeling ChR2. The specific four-state Markov model used here, **Figure 1B**, is loosely based on functional data about the channelrhodopsin photocycle kinetics [26], [27] and has been suggested as most suitable [9], [12], although not unique. The simpler three-state model structure, used in the model of ChR2-H134R by Abilez et al. [10], discussed above, is non-branching, with strictly unidirectional flow of transitions, unlike the four-state model used here. Despite its ability to capture qualitatively most of the ChR2 features, such circular three-state model has been criticized previously for limitations in the quantitative description of ChR2's light sensitivity, switch-off kinetics and pH-dependent action spectra shifts [9], [33]. Some of these limitations also may have contributed to the discrepancies with our model in **Figures S4–S7**.

All published mathematical models of ChR2 invariably describe the transition from C_2_ to C_1_ as irreversible, i.e. C_2_ will always recover completely to C_1_ in the dark. However, Ernst et al. detected in Volvox Channelrhodopsin (VChR) a pH-dependent equilibrium between photocycle dark states D470 and D480, which approximate to states C_1_ and C_2_ in our mathematical model of ChR2 [27], [33]. To probe for the existence of such dark-state equilibrium and its potential voltage-dependence in ChR2, we designed an experimental protocol (**Figure S8B**) with varied “pre-conditioning” voltage for an extended period in the dark prior to clamping at −80 mV and optically stimulating with blue light.

Our experimental results (**Figure S8C–F**) show an increase in peak current (I_P_), faster activation kinetics (τ_ON_) and slower kinetics of inactivation (τ_INACT_) with more positive pre-stimulation voltages, particularly above 0 mV. This suggests the presence of voltage-dependent dark equilibrium between C_1_ and C_2_ with increasing preference for the C_1_ state at more positive voltages. Therefore, an augmented 4-state, 8-transition model can be considered (**Figure S8A**), allowing for a C_1_→C_2_ transition with a rate G_r12_ (similar to e_12_ and e_21_), while the reverse transition is with a rate G_r21_.

In light of these results, the time constant for recovery from inactivation, τ_R_, corresponds to 1/(G_r12_+G_r21_) instead of the current τ_R_ = 1/G_r_ dependence. The development of a new model with such alternative structure can include the following constraints for determination of G_r12_(V) and G_r21_(V): 1) model output of peak current I_P_ and the ratio of peak current to steady-state current I_P_/I_SS_, 2) model activation kinetics (τ_ON_), and 3) G_r_(V), which slows with more positive voltages. Preliminary optimization attempts to accommodate such a model structure within these constraints and without introducing additional state transitions showed that a significant increase in γ (the ratio of O_2_/O_1_) from its current value of 0.1 is needed. The validity of such a change in the model is unclear, and several alternative scenarios are possible. Namely, a different set of constraints may yield more plausible model parameters. Alternatively, additional model transitions may be added as in Stehfest et al. [27] that will relax the constraints and possibly yield a different optimal parameter set. In either case, re-structuring the model requires more experimental data than provided here.

### Voltage dependencies of ChR2

The first incarnation of a channelrhodopsin model used a reaction scheme built around ChR1's photocycle [12] and voltage-dependent nonlinearities were ignored. This simplified view persisted in the follow-up model of ChR2 [9] which adopted the same state diagram and framework and kept the linear current-voltage relationship. To capture the prominent rectification properties of ChR2 with minimal outward current [6], [18], [20], [21], [30], [31], more recent models have introduced some empirically-derived nonlinearity [8], [10]. Similarly, our approach to modeling ChR2 rectification was purely empirical – we defined a well-behaved nonlinear function that best fit the experimental data, G(V) in **Table 1** **, Figure S1**. Alternatively, inward rectification can be modeled in a more mechanistic way if compelling experimental data are available. Recently, Gradmann et al. [31] discussed several possible mechanisms for the rectification properties of ChR2. From mathematical/biophysical point of view, these can involve: 1) classic Hodgkin-Huxley style gating; 2) Goldmann-Hodgkin-Katz mechanism of rectification; 3) asymmetric barrier/leaky proton pump – a mechanism put forward by Feldbauer et al. to explain rectification by single-channel properties from noise analysis [21]; and 4) a mechanism based on the kinetics of multiple ion species interacting with the channel. The last mechanism was favored in [31], whereas the remaining three were eliminated based on insufficient nonlinearity to match the experimental data.

In addition to the scenarios discussed in [31], we note that cytosolic Mg^2+^ and cytosolic polyamines (spermine, spermidine, putrescine, cadaverine) are known as common/universal mediators of ion current rectification across a variety of channels, although the exact mechanisms remain elusive. Mg^2+^ block and rectification was first reported in the 1980s for inward rectifier channels from the Kir family, including the cardiac Ik1(Kir2.1) [40], [41], as well as for ACh-sensitive K^+^ channels [42]. Later, it was established that Mg^2+^ acts at low-affinity sites, while endogenous cytosolic polyamines act on high-affinity sites and both mediate rectification. Importantly, polyamines' role in rectification was confirmed for a variety of target channels: inward rectifier, including the cardiac isoform (Kir2.1) by Lopatin and Ficker [43], [44], ryanodine receptors and their rectification properties [45], Ca^2+^ sensitive AMPA receptors [46], and recently also for rectification of cloned Na^+^ channels, especially the cardiac isoform (Nav1.5) [47]. Future work should test the possibility for polyamine-mediated rectification in ChR2; such new experimental data can form the basis for a more mechanistic description of voltage rectification within the ChR2 model.

The voltage dependencies in our ChR2 model also extend to some kinetic properties. More specifically, in order to match the experimental data, we had to introduce voltage dependence in the time constants associated with ChR2 opening, closing and especially recovery from inactivation, **Table 2**. The mechanism of such voltage sensitivity has not been discussed before. Very recent structural data on ChR2 [48], [49] provide only general information about the seven transmembrane domains, four of which (1, 2, 3 and 7) define the conductive pore, with TM2 determining its ion selectivity, and TM7 being critical for the interaction with the chromophore. We did not find direct evidence for a unique region within the ChR2 pore that is equivalent to a classical voltage sensor, i.e. a specific sequence of charged amino acids (Arg^+^, Lys^+^ residues at every third position with nonpolar residues in-between), normally found in the S4 transmembrane domain of voltage-gated ion channels with a tetrameric structure [50], see **Figure S9**. However, many ligand-gated ion channels, e.g. ACh channel, have been known to display voltage-dependent gating in the absence of classical voltage sensors, but purely due to basic electrostatic interactions between the charged ion channel pore and the flowing ions [50]. Further work (through mutagenesis) can pinpoint the mechanism of ChR2's voltage-dependent kinetics suggested by our data.

### Application of ChR2 model to optical excitation of cardiac tissue

With the realization that optogenetics implies the use of ChR2 within mammalian cells, including cardiac, at physiological temperatures, we derive here scaling factors (Q10) for the model parameters for accurate description of its operation at 37°C, **Figure 5**. We deem this essential since most characterization is done at room temperature, while within mammalian cells the operation of ChR2 will be critically dependent on its interaction with the endogenous ion channels, whose properties have been scaled to physiological temperatures. Interestingly, we find that Q10 scaling values for ChR2 parameters (**Figure 5**) fall within the range of temperature sensitivity values for classical voltage-gated ion channels [34], with expected acceleration of kinetics at elevated temperatures.

To gain confidence in the performance of the proposed model within a cardiac myocyte, it is important to validate the contribution of this exogenous current within the cardiac electrophysiology milieu. Such experimental data for I_ChR2_ during a cardiac action potential has not been presented before. Classically, to dissect ionic currents during an action potential, AP clamp approaches [36] are used in conjunction with specific pharmacological blocking agents. Lack of light is a very selective “blocker” of I_ChR2_, however enabling I_ChR2_ (within the AP clamp context) is more complicated. Owing to the dynamic response of I_ChR2_ to a light pulse (in contrast to the response of a typical ion channel to the presence of a blocker), a modified version of the AP clamp was used here with a precisely synchronized optical pulse to extract I_ChR2_. The overall excellent agreement between the model-predicted and experimentally-obtained I_ChR2_ during a guinea pig ventricular action potential (**Figure 6**) lends support to the use of this ChR2 model in other cell types. However, caution should be applied when longer-term optical pacing is considered since this model has not been experimentally constrained to reflect potential long-term inactivation, potential changes in the pH and/or the reversal potential.

Among the advantages of optogenetic stimulation are the spatiotemporal resolution and specificity of action, normally achieved by a cell-type specific promoter for gene expression. These advantages are currently at the crux of deciphering neural circuits in the brain [16]. Similarly, in the heart, there is cell type diversity and spatial gradients of ion channel expression, however, many fewer experimental tools are available for specific cell targeting, i.e. cell-specific promoters for driving exogenous gene expression. Alternative targeting of cell types can be achieved by dosing optical energy and taking advantage of the different excitation threshold of cardiomyocytes. **Figure 7** illustrates that, similar to electrical stimulation, optical excitation threshold is much lower for Purkinje cells than atrial or ventricular myocytes. Different ion channel composition resulting in altered balance of depolarizing (excitatory) and repolarizing (inhibitory) currents across cardiac cells plays an important role in their response to ChR2 as well. More specifically, the human cardiomyocyte models discussed here, have distinctly different inward rectifier current, I_K1_, which serves as the main opposing force to excitatory signals - peak I_K1_ after a 10 ms optical pulse for V: 1.84 pA/pF; for A: 0.49 pA/pF, and for P: 0.32 pA/pF (see also **Figure S10**). Indeed, when simulating a ventricular myocyte with a Purkinje formulation of I_K1_ (squares in **Figure 7C**), we find a significant reduction in excitation threshold (down to 12%, 28% and 37% of the original V-cell irradiance at 100 ms, 20 ms and 5 ms pulses, respectively. In addition to differences in single-cell electrophysiology, the coupling environment of these cell types is different, with ventricular myocytes facing the highest electrotonic load. These results suggest that the Purkinje cells (and the conduction system, in general) may be the preferential target for *in vivo* optical pacing at very low energy. We have confirmed this in a 3D computational heart model exploring different spatial arrangements [51].

Further utility of the ChR2 model developed here can be found in guiding the optimization of spatial gene and light delivery, as demonstrated in our preliminary simulations [51], [52] for *in vivo* low-energy use. Within cardiac optogenetics, the extension of this work to realistic heart geometries [29], [51] can precede and guide the design of proper optical actuating assemblies, which face many more challenges than the neuronal *in vivo* tools built around stereotactic systems. As the optogenetics toolbox expands to include more new mutants to serve different experimental needs, we believe that the computational framework presented here (with algorithms for automatic parameter identification) can be adopted to morph this model into other newly developed channels.

## Materials and Methods

### HEK-ChR2 cell line

A stable HEK cell line expressing ChR2-H134R-eYFP was developed by our group as described previously [18]. In short, the ChR2 plasmid (pcDNA3.1/hChR2(H134R)-EYFP, obtained through Addgene (Cambridge, MA) was used to transfect HEK 293 cells (ATCC, Manassas, VA) with Lipofectamine 2000 (Invitrogen, Carlsbad, CA) followed by 500 µg/mL of Geneticin (GIBCO Invitrogen) selection to achieve >98% expression.

### Experimental measurements of ChR2 current

Whole-cell patch clamp experiments were used to record the ChR2-mediated current. HEK-ChR2 cells were harvested by trypsinization and replated at a low density on polylysine-coated coverslips prior to experimentation. Cells were clamped using an Axopatch 1D amplifier (Axon Instruments Inc., Foster City, CA) and borosilicate glass pipettes (World Precision Instruments, Inc., Sarasota, FL) at room temperature (22°C). An inverted microscope (Olympus, CK40) was used for visualization during the patch-clamp procedure. Membrane currents were at recorded sampling frequencies of 200 Hz to 10 kHz (latter used for all kinetics measurements), digitized (DIGIDATA 1320A, Axon Instruments), and stored for subsequent offline analysis. The pipette solution contained (in mmol/L) potassium aspartate 80, KCl 50, MgCl_2_ 1, MgATP 3, EGTA 10, and HEPES 10 (pH 7.4 with KOH). The external solution contained (in mmol/L) KCl 5.4, NaCl 140, MgCl_2_ 1, CaCl_2_ 1.8, HEPES 10, and glucose 10 (pH 7.4 with NaOH).

Light-mediated ChR2 currents were triggered by a blue LED (470 nm) unit attached to the microscope epi-illumination port and controlled by a high-power LED driver (both from Thorlabs, Newton, NJ). Light was delivered to the cells through a 40× objective. Optical power was measured with a standard optical power meter (PMD100D, Thorlabs), and irradiance (mW/mm^2^) was calculated based on the illumination spot size (average, 0.78 mm^2^). Light pulses were synchronized with both the initiation of the voltage clamp and signal recording by a custom-designed interface via TTL to implement the experimental protocols shown in **Figure 1CD**.

ChR2 amplitude and kinetics were determined as shown in **Figure 1C**. Each cell (n = 7) was initially clamped to a holding voltage ranging from −10 to −80 mV. Ten ms post-voltage clamp, a 500 ms light pulse was delivered with irradiances ranging from 0.34 to 5.5 mW/mm^2^. Sufficient time (>10 s) was allowed between recording each combination of voltage and irradiance (total of 20 combinations per cell). Recovery from light-induced inactivation was explored using a second protocol shown in **Figure 2B**. Two consecutive 500 ms light pulses (S1 and S2) were applied, separated by a recovery interval (dark period). All combinations of holding voltages from −10 to −80 mV, irradiances of 0.44 to 3.44 mW/mm^2^, and recovery intervals of 0.5 to 15 s (a total of 60 combinations) were explored in the analyzed cells (n = 4).

### Data analysis: Filtering, segmentation and derivation of empirical parameters

All data analysis was completed offline in MATLAB (Mathworks, Natick, MA). Empirical parameters were extracted from each experimental trace as shown in **Figure 1A**. The time constants, τ_ON_, τ_INACT_, and τ_OFF_, respectively describe activation (light on), inactivation, and deactivation (light off) processes. Current magnitude was characterized by I_P_ (peak current) and I_SS_ (steady-state current). Median filtering was performed on all current traces prior to analysis. All curve fits were done with the MATLAB Curve Fitting Toolbox using the “trust-region” algorithm with variable tolerance of 10^−12^, minimum and maximum step size - 10^−12^ and 0.1, respectively; maximum number of 600000 iterations and function evaluations was used as a criterion for termination.

To determine time constants, ChR2 current traces were segmented into three parts and fit to mono-exponential curves. For each trace, I_P_ was determined to be the maximum (peak) current magnitude; the steady-state current, I_SS_, was calculated as the mean of current values 400 to 450 ms after the onset. Bounds and length of segmentation for each parameter were as follows: 1) for τ_ON_, 10 ms before I_P_ to I_P_; 2) for τ_INACT_, 10 to 110 ms after I_P_; 3) for τ_OFF_, 500 to 600 ms after I_P_. In addition, both I_P_ and I_SS_ were normalized to the baseline current, as well as cell capacitance.

To evaluate recovery from inactivation, percent recovery from inactivation was determined as the ratio of the peak current during the second vs. the first pulse (I_P1_/I_P2_). Mono-exponential curve fits were performed on data acquired from 1.6 mW/mm^2^ light pulses to find the curve fit time constant of recovery, τ_R_. In the absence of light, it follows from **Figure 1B** that G_r_≈1/τ_R_, thus a voltage-dependent exponential relationship was derived for the transition rate G_r_. In comparing experiment and model, τ_R_ was calculated for each combination of voltage and irradiance as the time at which I_P1_/I_P2_≈1−1/e≈0.632 after cubic spline interpolation as in **Figure 4A–D**.

### Statistical analysis of kinetic parameters

Since ChR2 kinetic parameters have not been previously considered to be both voltage and irradiance dependent, we performed statistical analysis on experimentally-derived τ_ON_, τ_OFF_ and τ_INACT_. Regression analysis was done in SPSS using a two-way ANOVA (with voltage and irradiance as factors), considering significance at P<0.01.

### ChR2 model implementation and parameter optimization

The implementation of ChR2 is based on the four-state model structure (**Figure 1B**), proposed earlier [9], [12], containing two open states (O_1_ and O_2_) and two closed states (C_1_ and C_2_), and modulated by a total of seven kinetic parameters (k_1_, k_2_, G_d1_, G_d2_, G_r_, e_12_, e_21_), see also **Table 1**. Model equations are presented below, **Eqns (1** **–** **14)**. Voltage dependence was introduced in G_V_, G_r_, and G_d1_ to reproduce the inward rectification and voltage-dependent kinetics found experimentally. Upon a fixed function form, model parameters were obtained via non-linear constrained optimization using a simulated annealing algorithm (Boltzmann type) in MATLAB, where the settings included an objective limit of 0, initial temperature of 100,000, reannealing interval of 50, and a maximum function evaluation limit of 1,000,000. Optimal parameter values determined by simulated annealing were used as starting values for patternsearch (greedy algorithm) for high-precision determination of local minima. Objective (error) function for parameter optimizations was determined as the root-mean-square (RMS) error of computational and experimentally-observed values of each parameter across voltages and irradiances.

The mathematical model of ChR2 was implemented in MATLAB with a robust protocol for electrical and optical stimulation. The modular form of the ChR2 current model allowed for direct plug-in into single-cell cardiac models, also implemented in MATLAB. Integration for the cell models was done using a built-in integration algorithm with a variable time step and suited for stiff systems of ordinary differential equations (ode15s) at absolute and relative error tolerances of 10^−10^. Cardiac cell models used in this study included a version of the Luo-Rudy model of a guinea pig ventricular cardiomyocyte [53], the 2006 version of the human ventricular cell model by ten Tusscher et al. [37], the human atrial model by Courtemanche et al. [38] and the Purkinje model by Sampson et al. [39]. The voltage output for the cardiac cell models has a general form, **Eqn (15)**, with the total current computed as described in the original references for guinea pig ventricular cells (*I_iLR_*), and for human ventricular (*I_iV_*), atrial (*I_iA_*) and Purkinje (*I_iP_*) cells, **Eqns (16** **–** **19)**.
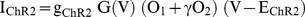
(1)

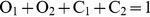
(2)

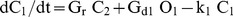
(3)

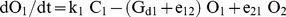
(4)

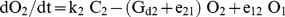
(5)

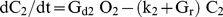
(6)where
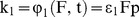
(7)

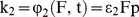
(8)

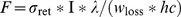
(9)

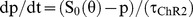
(10)

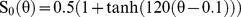
(11)

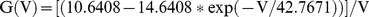
(12)

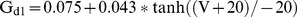
(13)

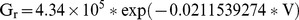
(14)

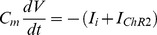
(15)

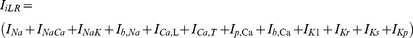
(16)

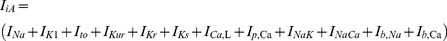
(17)

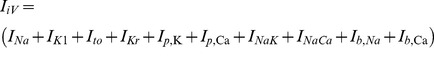
(18)

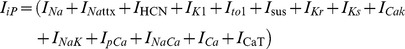
(19)


Strength-duration curves (such as shown in **Figure 7**) were constructed by varying optical pulse width and determining the minimum irradiance needed to excite. Threshold for excitation was defined as the stimulus duration and strength (irradiance) required to increase membrane voltage above −20 mV within 400 ms of stimulus initialization. The strength for each duration was computed by calculating error as [V_max_ – (−20)] and determining the value required to cross zero using the fzero function in MATLAB.

### Derivation of temperature scaling factors, Q_10_

Adjustment of parameter values for temperature conditions was done using a Q_10_ scaling. Q_10_ has been adopted in the literature on ion channels and excitable membranes as a simplified factor to correct temperature-dependent processes assuming Arrhenius (exponential) model of reaction kinetics. Using experimental data at 22°C and 37°C from [30] on four model parameters/constraints as shown in **Figure 5E**, we derived Q_10_ for each one of them, as follows:


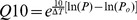
, where *P* and *P_o_* are the values of each of the four parameters (τ_ON_, τ_OFF_, τ_INACT_ and I_SS_/I_P_) measured at 37°C and at 22°C, respectively. Since the derived Q_10_ values were mostly voltage-independent, we used the averages as constraints. Simulated annealing was then applied, as described above, to derive Q_10_ values for the seven explicit model parameters (see **Figure 5E**), while minimizing the difference between model and experimental Q_10_ for the constraints. Finally, the values of the seven model parameters (obtained at 22°C) were scaled with the resultant optimal Q_10_s to represent the model behavior at physiological temperature.

### ChR2 current during the cardiac action potential (optical action potential clamp)

Experiments with optical excitation of cardiomyocytes to extract the ChR2 current during a cardiac action potential were done in virally-transduced adult guinea pig cells. For quick expression, adenoviral vector was constructed containing the transgene hChR2(H134R)-EYFP (based on plasmid no. 20940 from Addgene). Adult guinea pig left-ventricular cardiomyocytes were isolated as previously described [54], plated onto fibronectin-coated glass-bottomed dishes and incubated at 37°C in M199 media (Gibco) supplemented with 10 mM HEPES, 1% Pen/Strep, and 15% FBS. Viral infection was initiated three hours after plating. Viral particles were diluted in M199 media containing 2% FBS and applied to the cardiomyocytes during a two hour incubation period during which the dishes were gently shaken every 20 minutes to disperse the virus. Transgene expression was assessed 48 hours after exposure at which time ∼50% of the cells exhibited eYFP fluorescence (**Figure 6A**) and a measureable response to optical excitation.

Whole-cell patch clamp experiments were performed 40–48h post-infection using the setup and solutions described above for ChR2 measurements. ChR2-expressing cells were identified by the EYFP fluorescence. Barium (0.5 mmol/L) was added to the pipette solution to facilitate optical excitation by reducing the inward rectifier current. Timing synchronization between the LED and the patch-clamp software was done by computer control using a USB data acquisition controller (Measurement Computing, Norton, MA).

A version of the classic action potential clamp (AP clamp) approach [36] was used to extract the ChR2 current during a ventricular action potential. After confirming cell's electrical and optical excitability, an optically-triggered (470 nm, 50 ms, 1.5 mW/mm^2^) action potential was recorded under current clamp mode (I = 0) for later use in AP clamp regime. The optical AP waveform was then used in a voltage-clamp mode under two different conditions and the total resultant current was recorded. First, the AP clamp was applied in the dark, thus obtaining a total ionic current without the ChR2 contribution. Second, the AP clamp was applied in conjunction with a light pulse, precisely timed with respect to the clamping AP (to replicate the conditions of the original AP record) and the total current containing ChR2 contribution was saved. The difference between these two total currents (net current, normalized by the cell capacitance) was interpreted as the ChR2 current (**Figure 6B–D**). Upon AP clamp application, immediately before the light pulse, the cell was pre-clamped to 0 mV to temporarily reduce availability of sodium channels and therefore avoid interference by this large current during the AP upstroke.

## Supporting Information

Figure S1 **Empirical voltage-dependent functions in the model.** **A**. Dimensionless rectification function G(V); **B**. Rate function G_d1_(V); **C**. Rate function G_r_(V). Plots are based on the equations listed in **Table 1**.
(TIF)

Figure S2 **ChR2 gene expression (current density) levels and effects on optical excitability.** Shown are log-log plots for the irradiance levels needed to excite at four different pulse durations for ventricular (**A**), atrial (**B**) and Purkinje cells (**C**). Conductance scaling of 1 is the current (default) value used in the model.
(TIF)

Figure S3 **Effects of long-term fast optical pacing on state occupancy and availability of ChR2 current.** Shown are the state occupancies for O1, O2, C1 and C2 (**A–D**) and the total ChR2 current (**E**) for the first and last optically paced beat in a ventricular myocyte upon 100 sec of 2 Hz pacing with 10 ms pulses at 1 mW/mm^2^.
(TIF)

Figure S4 **Comparison between two models of ChR2 with and without voltage dependencies.** Comparison between the current Stony Brook model with voltage dependences, *SB(V)*, and an equivalent 4-state model with no voltage dependences (no rectification and no voltage-dependent kinetics), *noV*. **A**. Current-voltage relationships for a fixed irradiance of 1 mW/mm^2^; **B**. example current responses to a 500 ms light pulse at 1 mW/mm^2^ and a holding voltage of −40 mV (as indicated by circles in panel A. **C** and **D**. Response of a ventricular cardiomyocyte to a 200 ms light pulse at 1 mW/mm^2^ – shown are the respective action potentials (**C**) and underlying ChR2 currents (**D**).
(TIF)

Figure S5 **Comparison between two models of ChR2-H134R: Voltage- and light- sensitivity** for the current Stony Brook 4-state model, *SB(4s)*, and the Stanford 3-state model, *S(3s)*, both modeling ChR2-H134R. **A–D**. Current responses to a 500 ms light pulses at irradiance of 1 or 5.5 mW/mm^2^ and a holding voltage of −80 or −20 mV (as indicated in each panel).
(TIF)

Figure S6 **Comparison between two models of ChR2-H134R: Steady-state to peak current ratio (I_ss_/I_P_) and time constant of onset (**τ**_ON_)** between the current Stony Brook 4-state model, *SB(4s)*, and the Stanford 3-state model, *S(3s)*. Dependence of I_ss_/I_P_ on irradiance (**A**), and on voltage (**B**). Dependence of τ_ON_ on irradiance (**C**), and on voltage (**D**). Conditions of the measurements indicated in the lower left corner for each panel, i.e. fixed voltage of −80 mV or fixed irradiance of 1 mW/mm^2^.
(TIF)

Figure S7 **Comparison between the response of a ventricular cell modified by two versions of ChR2-H134R -** the current Stony Brook 4-state model, *SB(4s)*, and the Stanford 3-state model, *S(3s)*. **A–B**. Optically-triggered action potentials in a ventricular myocyte (represented by the tenTusscher model) using a 200 ms light pulse at 5.5 mW/mm^2^ (**A**) and at 1 mW/mm^2^ (**B**). Insets show the underlying ChR2 current during the light pulse.
(TIF)

Figure S8 **Empirically probing for an alternative ChR2 model structure including dark-state equilibrium.** **A**. Schematic representation of an alternative ChR2 model structure including dark-state equilibrium (C_1_↔C_2_), and the possibility of that equilibrium to be voltage-dependent. **B**. Experimental protocol to test for the existence of dark-state equilibrium with pre-conditioning at different voltages and a brief (5 ms) reset to a common voltage (−80 mV) before the application of a light pulse. **C**. Example current traces from a cell subjected to the protocol shown in (**B**) – larger current is observed if the cell has been preconditioned with a more positive voltage. **D–F**. Summary of experimental data on peak current (I_P_), sustained current (I_SS_) and peak-sustained current ratio (I_P_/I_SS_) over the range of applied pre-conditioning voltages and for two irradiance levels. More positive pre-pulse voltage resulted in larger peak and sustained current as well as larger ration of peak-to-sustained current, and this voltage dependence was enhanced by higher irradiance levels. Data are shown as mean±S.E., n = 5.
(TIF)

Figure S9 **Comparison of amino-acid sequences for a classical voltage-sensing motif and ChR2.** **A**. Homology of classical voltage-sensing motifs among voltage-gated ion channels (after B. Hille), Arg+ (R) and Lys+ (K) residues are in red. **B**. Amino-acid sequence for the C1C2 chimera (Kato et al.) with the essential pore structure, and the full ChR2 sequence.
(EPS)

Figure S10 **Differential contribution of I_K1_ (opposing force to IChR2) in cardiac cell types.** Shown are I_K1_ traces during optically-induced action potentials in ventricular, atrial and Purkinje cells, using 10 ms pulses at 1 mW/mm^2^.
(TIF)

Text S1 **Analysis of cell heating by light.**
(DOCX)
