# Supplementary figures and images for: Computational Optogenetics: Empirically-Derived Voltage- and Light-Sensitive Channelrhodopsin-2 Model
Source: PLoS Comput Biol. 2013 Sep 12;9(9):e1003220. doi: 10.1371/journal.pcbi.1003220 (PMC3772068; doi:10.1371/journal.pcbi.1003220)

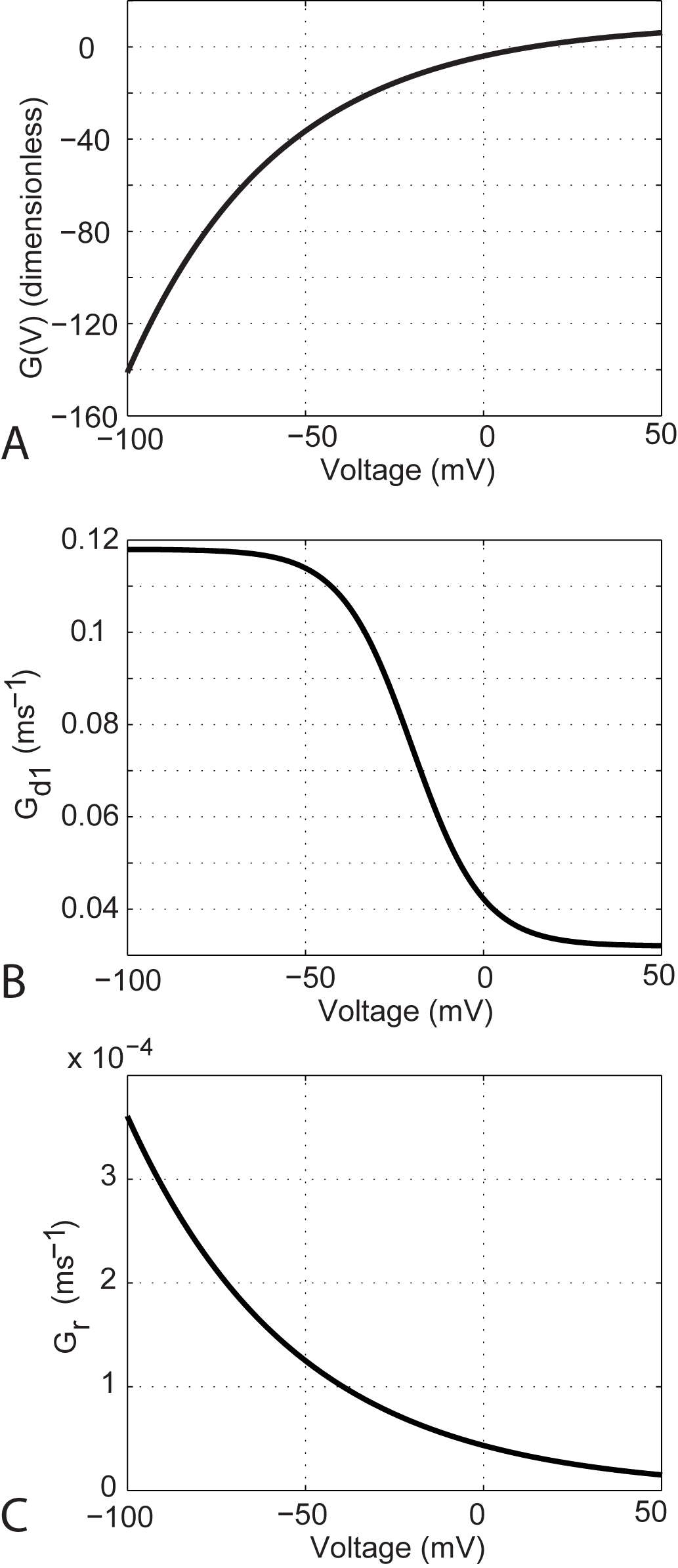

Supplement: Figure S1 — Empirical voltage-dependent functions in the model. A. Dimensionless rectification function G(V); B. Rate function Gd1(V); C. Rate function Gr(V). Plots are based on the equations listed in Table 1 . (TIF) [file pcbi.1003220.s001.tif]

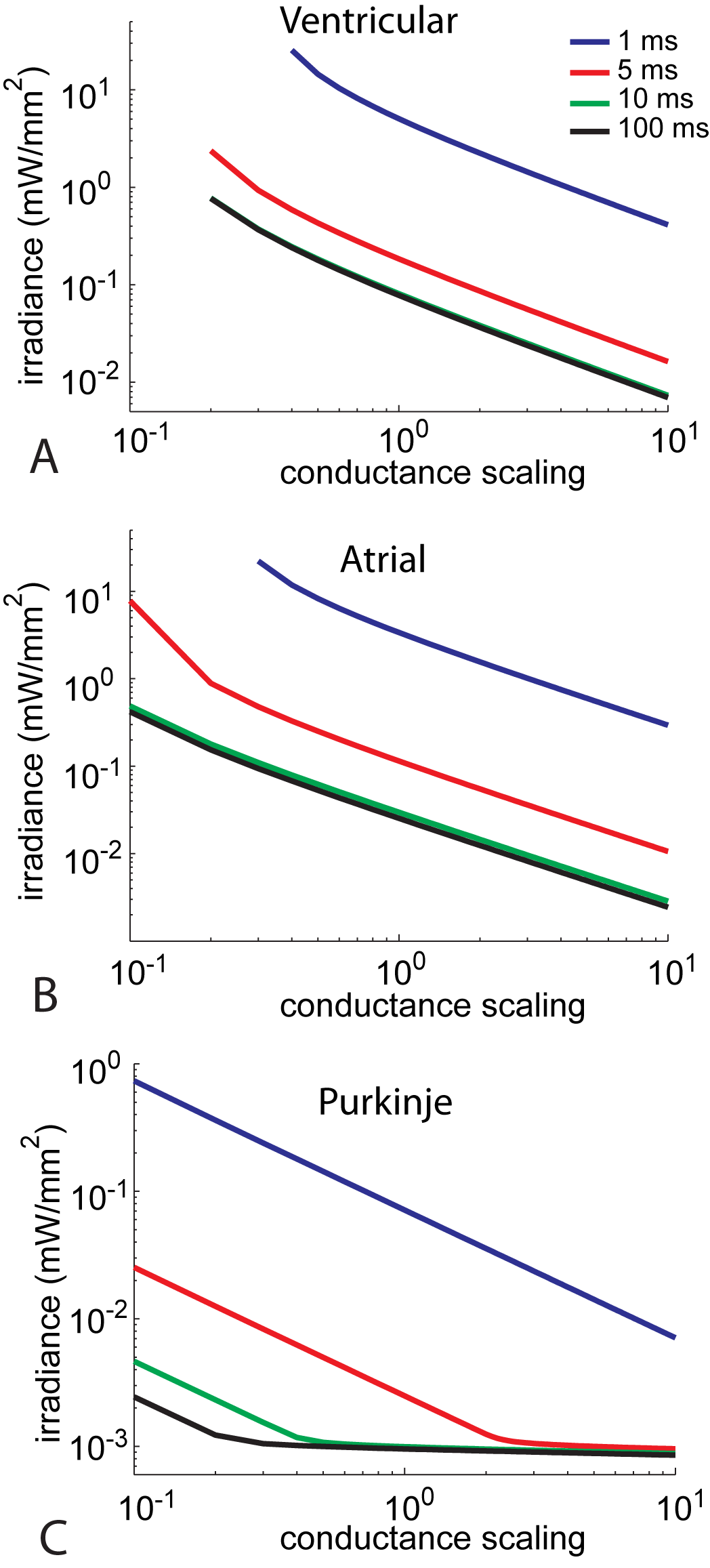

Supplement: Figure S2 — ChR2 gene expression (current density) levels and effects on optical excitability. Shown are log-log plots for the irradiance levels needed to excite at four different pulse durations for ventricular (A), atrial (B) and Purkinje cells (C). Conductance scaling of 1 is the current (default) value used in the model. (TIF) [file pcbi.1003220.s002.tif]

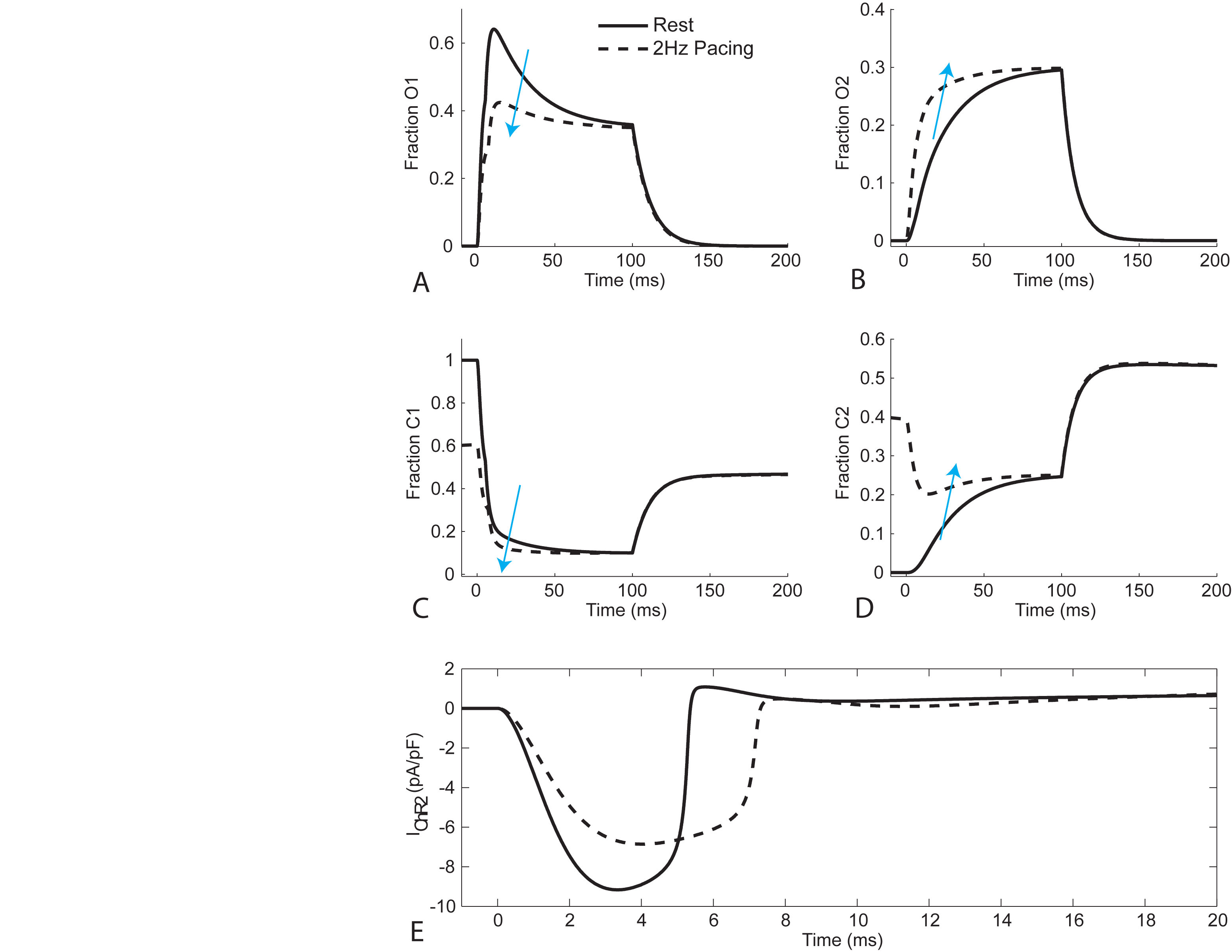

Supplement: Figure S3 — Effects of long-term fast optical pacing on state occupancy and availability of ChR2 current. Shown are the state occupancies for O1, O2, C1 and C2 (A–D) and the total ChR2 current (E) for the first and last optically paced beat in a ventricular myocyte upon 100 sec of 2 Hz pacing with 10 ms pulses at 1 mW/mm2. (TIF) [file pcbi.1003220.s003.tif]

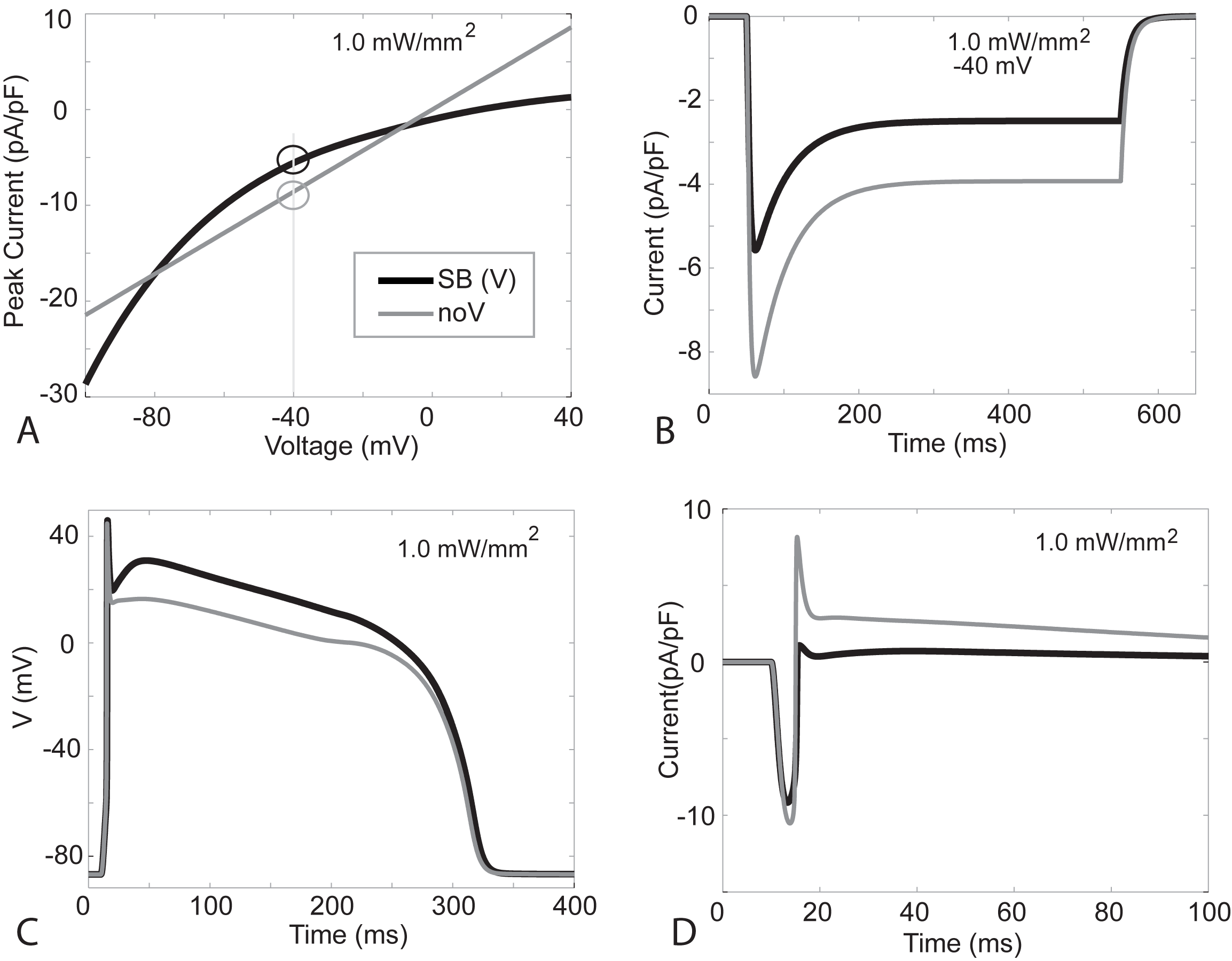

Supplement: Figure S4 — Comparison between two models of ChR2 with and without voltage dependencies. Comparison between the current Stony Brook model with voltage dependences, SB(V), and an equivalent 4-state model with no voltage dependences (no rectification and no voltage-dependent kinetics), noV. A. Current-voltage relationships for a fixed irradiance of 1 mW/mm2; B. example current responses to a 500 ms light pulse at 1 mW/mm2 and a holding voltage of −40 mV (as indicated by circles in panel A. C and D. Response of a ventricular cardiomyocyte to a 200 ms light pulse at 1 mW/mm2 – shown are the respective action potentials (C) and underlying ChR2 currents (D). (TIF) [file pcbi.1003220.s004.tif]

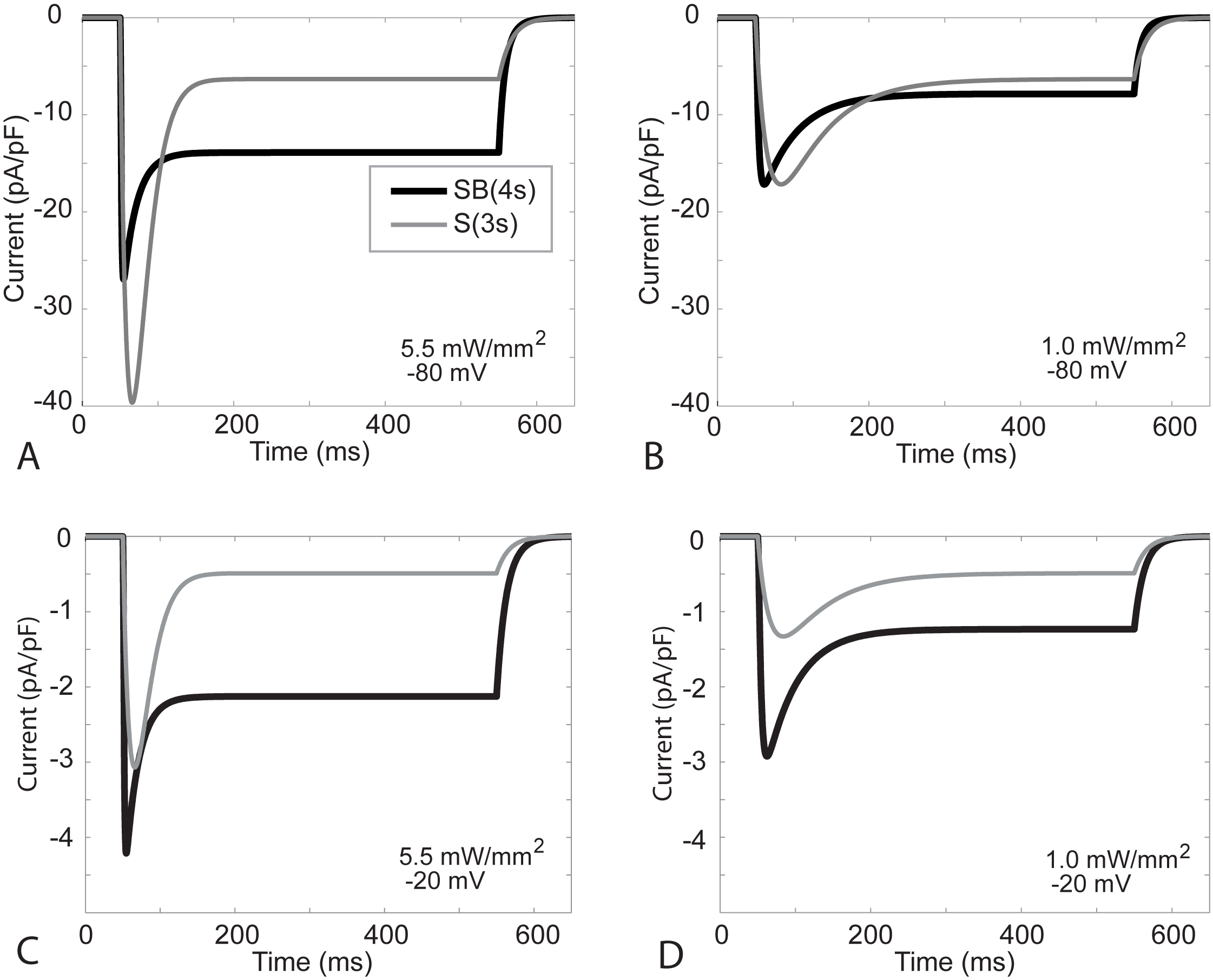

Supplement: Figure S5 — Comparison between two models of ChR2-H134R: Voltage- and light- sensitivity for the current Stony Brook 4-state model, SB(4s), and the Stanford 3-state model, S(3s), both modeling ChR2-H134R. A–D. Current responses to a 500 ms light pulses at irradiance of 1 or 5.5 mW/mm2 and a holding voltage of −80 or −20 mV (as indicated in each panel). (TIF) [file pcbi.1003220.s005.tif]

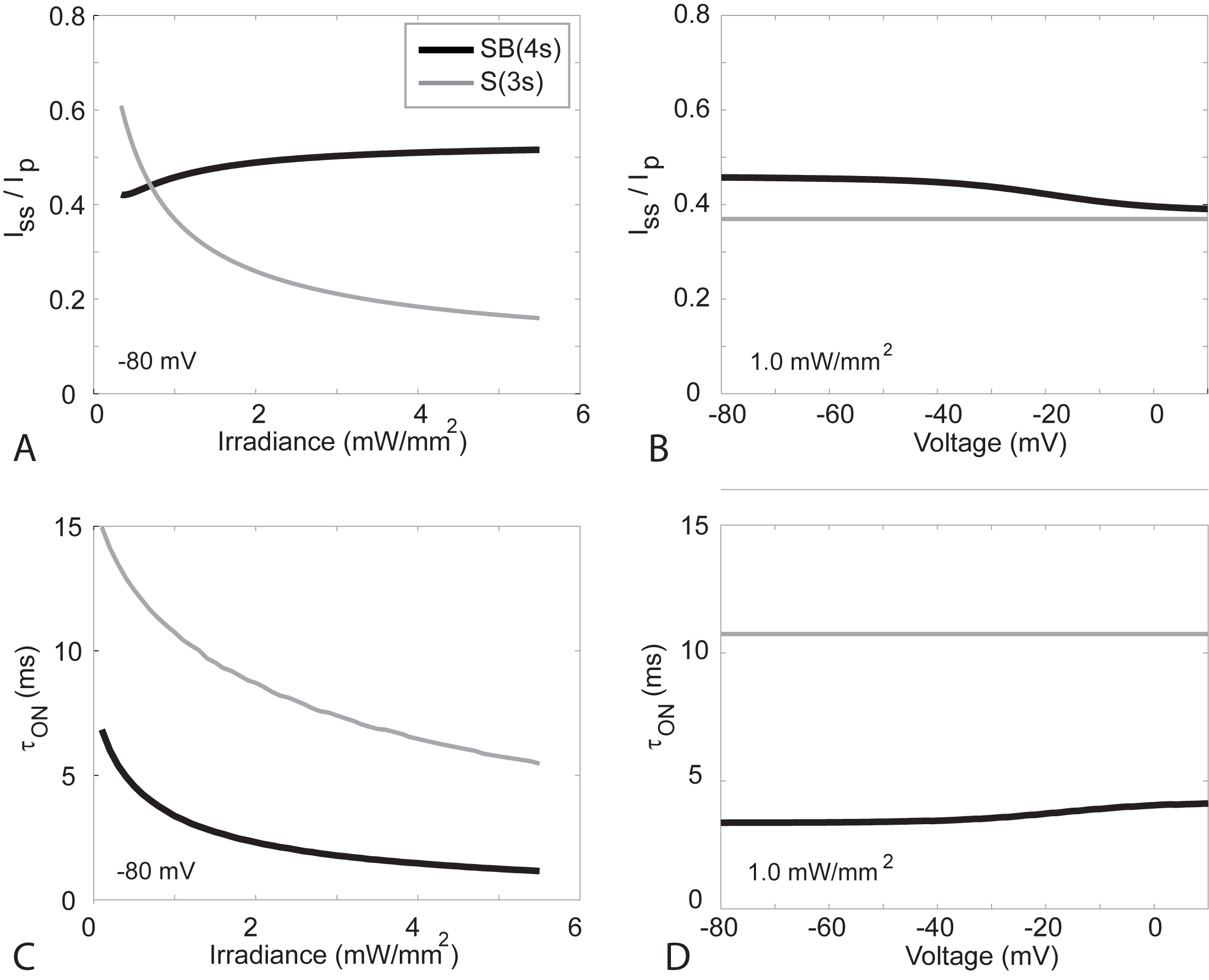

Supplement: Figure S6 — Comparison between two models of ChR2-H134R: Steady-state to peak current ratio (Iss/IP) and time constant of onset (τON) between the current Stony Brook 4-state model, SB(4s), and the Stanford 3-state model, S(3s). Dependence of Iss/IP on irradiance (A), and on voltage (B). Dependence of τON on irradiance (C), and on voltage (D). Conditions of the measurements indicated in the lower left corner for each panel, i.e. fixed voltage of −80 mV or fixed irradiance of 1 mW/mm2. (TIF) [file pcbi.1003220.s006.tif]

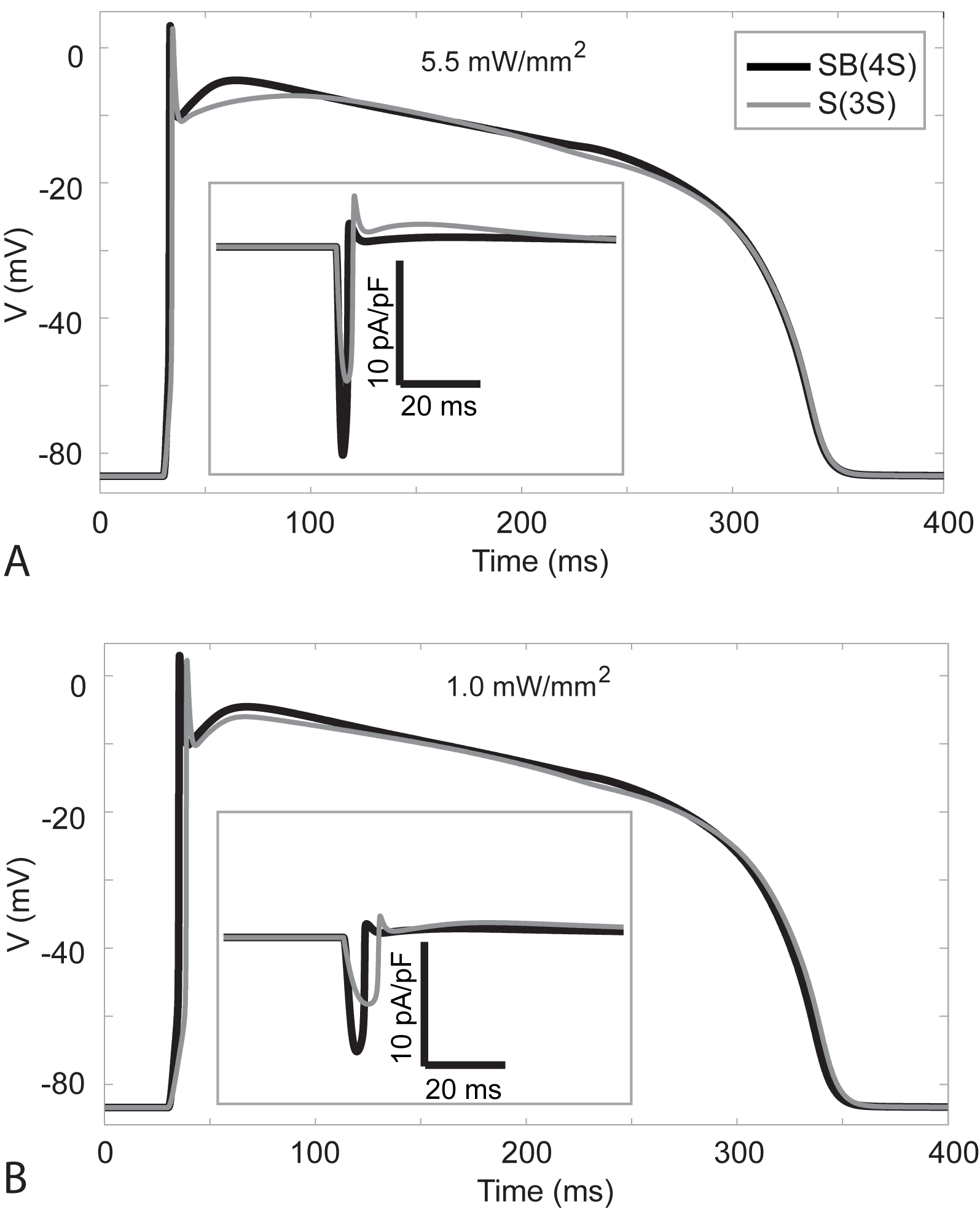

Supplement: Figure S7 — Comparison between the response of a ventricular cell modified by two versions of ChR2-H134R - the current Stony Brook 4-state model, SB(4s), and the Stanford 3-state model, S(3s). A–B. Optically-triggered action potentials in a ventricular myocyte (represented by the tenTusscher model) using a 200 ms light pulse at 5.5 mW/mm2 (A) and at 1 mW/mm2 (B). Insets show the underlying ChR2 current during the light pulse. (TIF) [file pcbi.1003220.s007.tif]

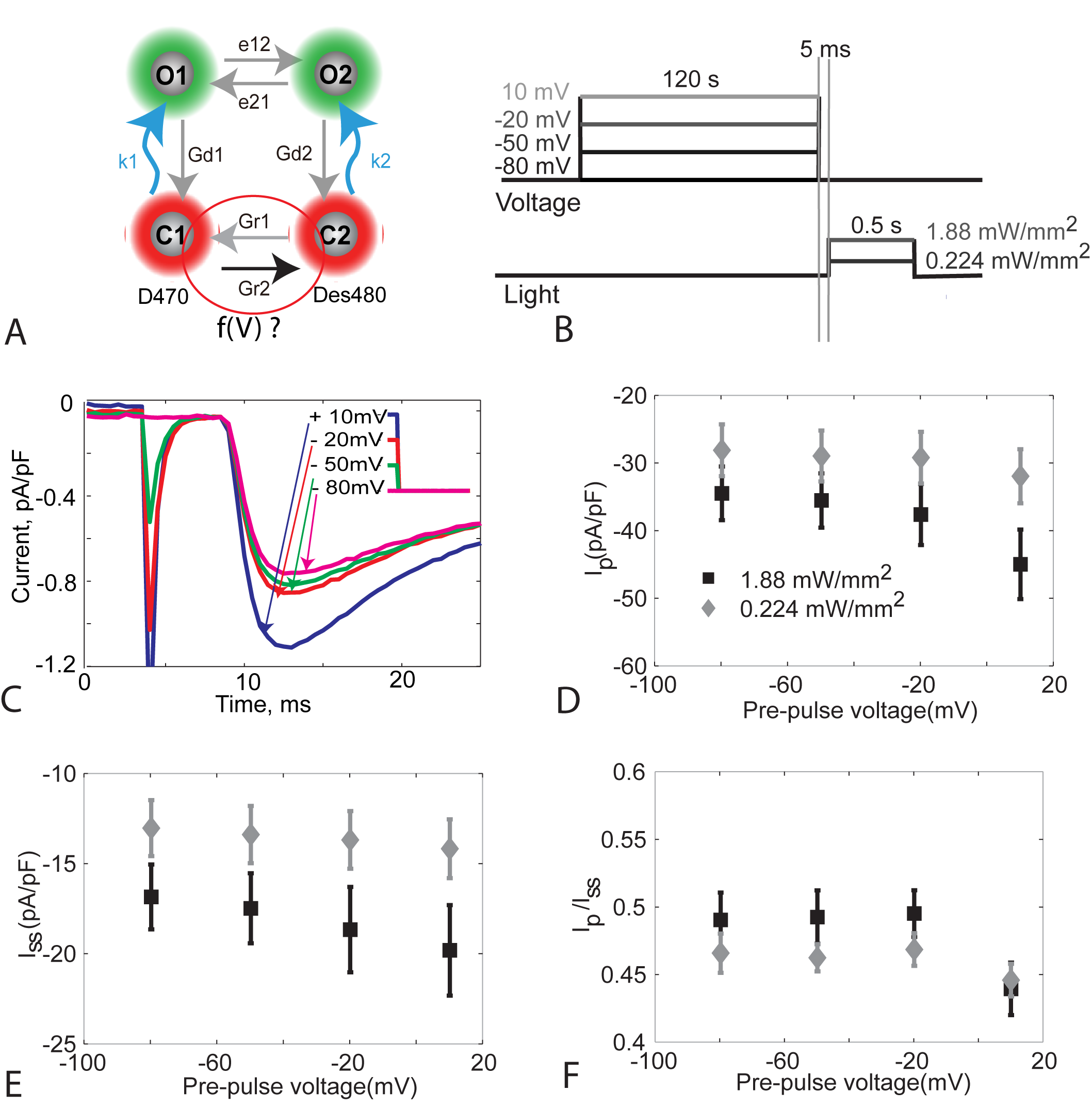

Supplement: Figure S8 — Empirically probing for an alternative ChR2 model structure including dark-state equilibrium. A. Schematic representation of an alternative ChR2 model structure including dark-state equilibrium (C1↔C2), and the possibility of that equilibrium to be voltage-dependent. B. Experimental protocol to test for the existence of dark-state equilibrium with pre-conditioning at different voltages and a brief (5 ms) reset to a common voltage (−80 mV) before the application of a light pulse. C. Example current traces from a cell subjected to the protocol shown in (B) – larger current is observed if the cell has been preconditioned with a more positive voltage. D–F. Summary of experimental data on peak current (IP), sustained current (ISS) and peak-sustained current ratio (IP/ISS) over the range of applied pre-conditioning voltages and for two irradiance levels. More positive pre-pulse voltage resulted in larger peak and sustained current as well as larger ration of peak-to-sustained current, and this voltage dependence was enhanced by higher irradiance levels. Data are shown as mean±S.E., n = 5. (TIF) [file pcbi.1003220.s008.tif]

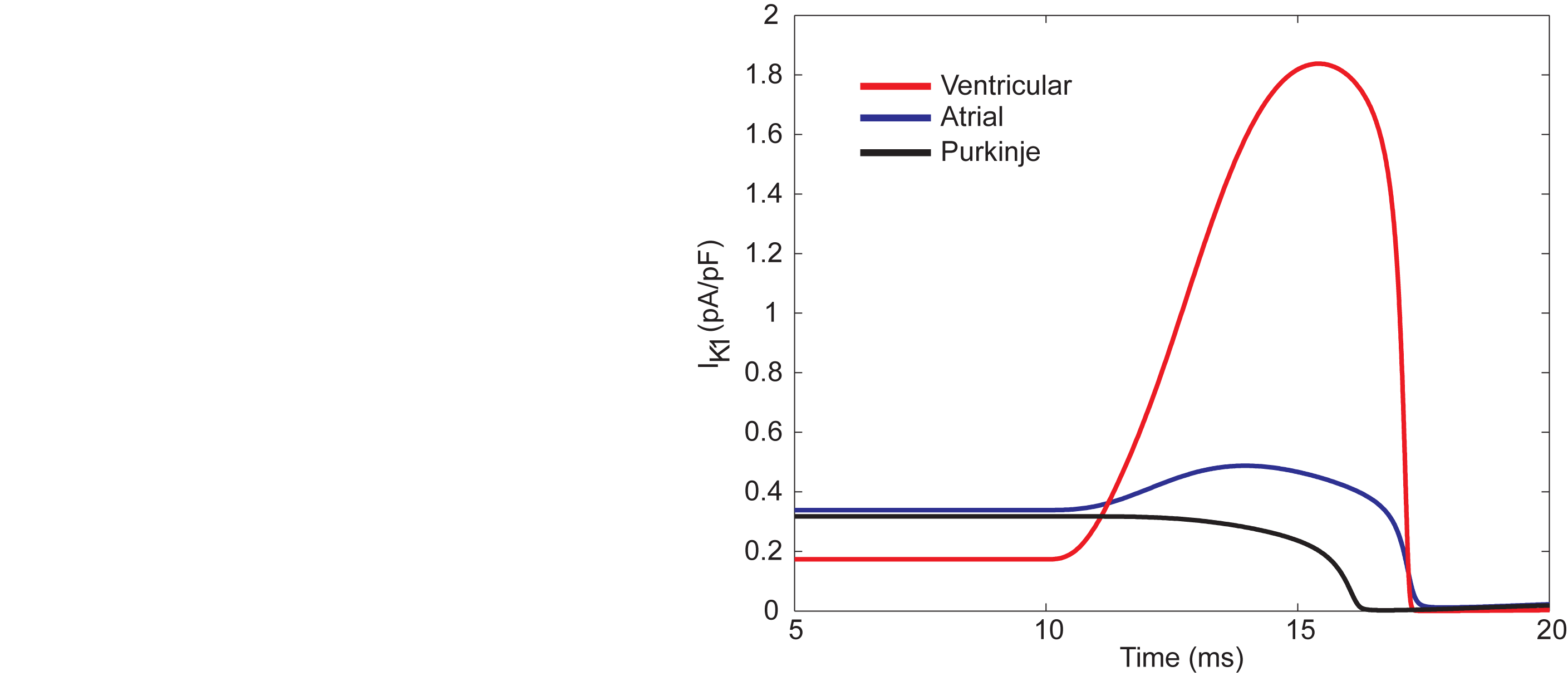

Supplement: Figure S10 — Differential contribution of IK1 (opposing force to IChR2) in cardiac cell types. Shown are IK1 traces during optically-induced action potentials in ventricular, atrial and Purkinje cells, using 10 ms pulses at 1 mW/mm2. (TIF) [file pcbi.1003220.s010.tif]
