# Supplementary material for: Computational Optogenetics: Empirically-Derived Voltage- and Light-Sensitive Channelrhodopsin-2 Model
Source: PLoS Comput Biol. 2013 Sep 12;9(9):e1003220. doi: 10.1371/journal.pcbi.1003220 (PMC3772068; doi:10.1371/journal.pcbi.1003220)
Supplement: Text S1 — Analysis of cell heating by light. (DOCX) [file pcbi.1003220.s011.docx]

**SUPPLEMENTARY INFORMATION**

1. **Analysis of Cell Heating by Light:**

We use the diffusion partial differential equation to describe the heating of a water-based particle within an aqueous medium:

$$\text{κ}\nabla^{2}T-\frac{\partial T}{\partial t}=Q\left(\vec{r}, t\right),$$

where $T$ is the sample temperature, $\kappa$ is the thermal diffusivity constant ($\kappa_{Water}= 1.43\times10^{-7} \frac{m^{2}}{s}$), and $Q\left(\vec{r}, t\right)$ is the rate of local heating given by[[1-4](#_ENREF_1)]

$$Q\left(\vec{r},t\right)=\frac{2\alpha P}{\pi w^{2}\rho c}e^{-\frac{2\vec{r}^{2}}{w^{2}}}=\frac{q}{\rho c}e^{-\frac{2\vec{r}^{2}}{w^{2}}}$$

where $q=2\alpha P/\pi w^{2}$, $\alpha$ is the absorption coefficient of the media ($\alpha_{Water}= 0.612 m^{-1 }@480nm$), $P$ is the incident light power, $w$ is the radius of the focused incident light, $\vec{r}$ is the radial distance from the axis of light propagation, $\rho$ is the density of the media ( $\rho_{Water}=10^{3}\frac{kg}{m^{3}}$), and $c$ is the specific heat of the media ($c_{water}=4182\frac{J}{kg K}$).

Because we are interested in the maximum local heating, we can set $\vec{r}=0$, as the maximum light intensity is at the center of the focused beam. Then, the closed-form solution of the above differential equation is:

$$T\left(t\right)=\frac{qw}{\rho c\kappa\sqrt{8\pi}}\left[\frac{\pi}{2}-\tan^{-1}\frac{w}{2\sqrt{2\kappa t}}\right]$$

**References:**

1. Axelrod D (1977) Cell surface heating during fluorescence photobleaching recovery experiments. Biophys J 18: 129-131.

2. Pope RM, Fry ES (1997) Absorption spectrum (380-700 nm) of pure water. II. Integrating cavity measurements. Appl Opt 36: 8710-8723.

3. Ebbesen CL, Bruus H (2012) Analysis of laser-induced heating in optical neuronal guidance. J Neurosci Methods 209: 168-177.

4. Liu Y, Cheng DK, Sonek GJ, Berns MW, Chapman CF, et al. (1995) Evidence for localized cell heating induced by infrared optical tweezers. Biophys J 68: 2137-2144.
